# An RXLR effector disrupts vesicle trafficking at ER-Golgi interface for *Phytophthora capsici* pathogenicity

**DOI:** 10.1016/j.mocell.2024.100158

**Published:** 2024-11-20

**Authors:** Jihyun Kim, Jesse Kaleku, Haeun Kim, Minji Kang, Hui Jeong Kang, Jongchan Woo, Hongshi Jin, Seungmee Jung, Cécile Segonzac, Eunsook Park, Doil Choi

**Affiliations:** 1 Department of Agriculture, Forestry and Bioresources, Plant Genomics and Breeding Institute, College of Agriculture and Life Science, Seoul National University, Seoul 08826, Republic of Korea; 2 Plant Immunity Research Center, College of Agriculture and Life Science, Seoul National University, Seoul 08826, Republic of Korea; 3 Department of Molecular Biology, College of Agricultural, Life Sciences and Natural Resources, University of Wyoming, WY 82071, USA; 4 Innocorelix, Seoul 07572, Republic of Korea

**Keywords:** ER stress, *Phytophthora capsici*, Rab small GTPase, RXLR effector, Vesicle trafficking

## Abstract

*Phytophthora* species, an oomycete plant pathogen, secrete effectors into plant cells throughout their life cycle for manipulating host immunity to achieve successful colonization. However, the molecular mechanisms underlying effector-triggered necrotic cell death remain elusive. In this study, we identified an RXLR (amino acid residue; Arginine-Any amino acid-Leucine-Arginine motif) effector (Pc12) from *Phytophthora capsici*, which contributes to virulence and induces necrosis by triggering a distinct endoplasmic reticulum (ER) stress response through its interaction with Rab13-2. The necrotic cell death induced by Pc12 did not exhibit conventional effector-triggered immunity-mediated hypersensitive cell death, including the involvement of nucleotide-binding site leucine-rich repeat downstream signaling components and transcriptional reprogramming of defense-related genes. Instead, it alters the localization of ER-resident proteins and confines secretory proteins within the ER. Pc12 directly interacts with Rab13-2, which is primarily localized to the ER and Golgi apparatus, resulting in a diminished Rab13-2 signal on the Golgi apparatus. Furthermore, Rab13-2 exhibits increased affinity for its interactor, Rab escort protein 1, in the presence of Pc12. Structural predictions revealed that a specific residue of Rab13-2 is crucial for binding to the C-terminus of Pc12. Substitution of this residue reduced its interaction with Pc12 and impaired *P. capsici* infection while maintaining its interaction with Rab escort protein 1 and prenylated Rab acceptor 1. These findings provide insight into how a pathogen effector induces a distinct form of necrotic cell death to facilitate colonization of the host plant by disrupting the recycling of Rab13-2, a protein involved in vesicle trafficking at the ER-Golgi interface.

## INTRODUCTION

*Phytophthora capsici* is an oomycete pathogen causing blight disease in economically important crops, including Solanaceae, Cucurbitaceae, Fabaceae, and Malvaceae, making it the fifth most destructive oomycete globally (Kamoun et al., 2015, Parada-Rojas et al., 2021, Quesada-Ocampo et al., 2023). *P. capsici* has a hemibiotrophic life cycle, switching from a biotrophic phase, in which it strategically establishes itself within the host plant, to a necrotrophic phase, which aims to extract nutrients from dead cells and release spores for subsequent dispersal (Kamoun et al., 2015, Parada-Rojas et al., 2021, Quesada-Ocampo et al., 2023). *P. capsici* secretes effectors to facilitate its successful colonization throughout infection phases, particularly during the necrotrophic phase, where some effectors induce necrosis to benefit the pathogen. For example, the necrotrophic fungus *Cochliobolus victoriae* secretes the fungal toxin victorin, which activates the defense protein LOV1 in *Arabidopsis*, thereby exploiting the plant’s defense mechanisms to enhance disease susceptibility (Huysmans et al., 2017, Lorang et al., 2012). *Phytophthora* species secrete RXLR (amino acid residue; Arginine-Any amino acid-Leucine-Arginine motif) effector proteins, characterized by a conserved Arg-X-Leu-Arg (RXLR) motif essential for their transport into host cells for manipulating plant defense responses to facilitate the pathogen’s successful colonization (Boevink et al., 2020, Fan et al., 2018, Kim et al., 2023, Wawra et al., 2017, Whisson et al., 2007). Fifty-two RXLR effectors from *Phytophthora infestans* have been found to localize at diverse subcellular compartments within plant cell, including the nucleus, cytoplasm, plasma membrane, other membranes, and the cytoskeleton (Wang et al., 2019). These effectors eventually target to various host factors, identified through proteomics screening, underscoring vesicle trafficking as a primary effector-targeted process (Petre et al., 2021). Vesicle trafficking is used to secrete plant defense-related proteins and hydrolytic enzymes such as pathogenesis-related proteins to defeat invading pathogens (van Loon et al., 2006, Wang et al., 2020). Several effectors from various pathogens are known to suppress the secretion of antimicrobial proteins such as PR1 and PDF1.2 or disrupt vesicle movement toward the pathogen’s focal area by targeting Rab proteins (Li et al., 2022, Pandey et al., 2021, Tomczynska et al., 2018). Thus, effective disruption of vesicle trafficking in plants is crucial for pathogen proliferation.

In intracellular vesicle trafficking, small GTPase Rab proteins function as key regulators of endomembrane vesicle dynamics in eukaryotic cells (Gray et al., 2020; Nielsen et al., 2008; Pylypenko et al., 2018; Stenmark, 2009). Within a single species, Rab proteins exist in multiple isoforms, each defining membrane identity and mediating vesicle trafficking to specific subcellular compartments (Gray et al., 2020, Nielsen et al., 2008, Pylypenko et al., 2018 Stenmark, 2009). Dysfunctional Rab proteins in vesicle trafficking can result in endoplasmic reticulum (ER) stress due to improper protein compartmentation (Kiral et al., 2018). The ER functions as a cellular factory responsible for protein synthesis, folding, and export to subcellular organelles. These processes must be carefully balanced to maintain cellular homeostasis under varying environmental conditions (Adams et al., 2019, Angelos et al., 2017, Cao and Kaufman, 2012). When this balance is disrupted, leading to the accumulation of misfolded or unfolded proteins, the ER undergoes stress and initiates the unfolded protein response (UPR). The UPR involves the translocation of transcription factors into the nucleus to activate genes essential for protein folding and ER-associated degradation (Adams et al., 2019, Angelos et al., 2017, Cao and Kaufman, 2012). However, under severe or chronic stress, plants may undergo unresolved ER stress-induced programmed cell death as a survival mechanism (Liu and Howell, 2016, Simoni et al., 2022, Yang et al., 2021, Yang et al., 2014).

In this study, we reported the RXLR effector Pc12 (also named CRISIS7 in Seo et al., 2022) from *P. capsici*, which induced necrotic cell death in the Solanaceae family. Pc12 enhanced the virulence of *P. capsici* in *Nicotiana benthamiana* with disrupting vesicle trafficking between the ER and Golgi apparatus, leading to the mislocalization of ER-resident proteins and the retention of secretory proteins. This disruption induced ER stress, accompanied by distinct patterns of UPR gene expression. Pc12 specifically targeted Rab13-2, causing its mislocalization from the Golgi apparatus and increasing its affinity for Rab escort protein 1 (REP1), a protein involved in Rab protein recycling.

## MATERIALS AND METHODS

### Plant Materials and Growth Conditions

*N. benthamiana, Nicotiana tabacum* cv*.* Samsun, and *Solanum lycopersicum* cv*.* Heinz seeds were directly sown into damp horticultural bed soil (Biogreen, Seoul, Korea) and cultivated within a walk-in chamber at 22°C to 24℃ under a 16-h/8-h (day/night) cycle. *Capsicum annuum* cultivar Early Calwonder (ECW) seeds were sterilized for 1 min in a 0.1% sodium hypochlorite solution and germinated in darkness at 30℃ for 7 days. Following this, the pepper seedlings were transplanted into soil and grown within the same chamber. For virus-induced gene-silencing assays, 3-week-old *N. benthamiana* plants were utilized. Transgenic *N. benthamiana* plants expressing Red Fluorescent Protein (RFP) fused to HDEL [amino acid residues; Histidine (H), Aspartic acid (D), Glutamic acid (E), and Leucine (L)] (ER marker, Chakrabarty et al., 2007) and GFP-Rab13-2 transgenic plants were purchased from the Nicotiana Genetic Stock Center. Plants were grown at 22°C to 24°C for 4 to 5 weeks prior to the corresponding experiments.

### *P. capsici* Culture Condition and Inoculation Assays

*P. capsici* strain 40,476 was cultured on V8 agar medium for 7 days at 23℃ in darkness. The mycelia were scraped and then incubated under light for 12 h. To induce the release of zoospores, sporangia were harvested and placed in distilled water, incubating at 4℃ for 30 min, followed by 23℃ for 30 min. A total of 500 zoospores were inoculated on the detached *N. benthamiana* leaves through droplets. The leaves were placed at 25℃ for 2 days under dim light. The lesion areas were measured at 2 days post inoculation.

### Constructs and Markers Preparation

RXLR effector and its mutants were amplified, incorporating an N-terminal 3xHA epitope excluding signal peptide and RXLR-EER (amino acid residue; Arginine-Any amino acid-Leucine-Arginine motif-Glutamic acid-Glutamic acid-Phenylalanine motif) motifs. Rab proteins and REP1 or PRA were amplified using cDNA of *N. benthamiana* and overlapped with N-terminal Green Fluorescent Protein (GFP) and 3xFLAG, respectively (Kim et al., 2017). Gene-of-interests were inserted into pCambia2300-LIC vector using the ligation-independent cloning method (Oh et al., 2010). RFP and RFP-Pc12 were amplified with the attB site to insert into an ethanol-inducible expression vector using Gateway cloning (Invitrogen, USA). The primer sequences used for construction are presented in Table S5. mCherry targeted to cis-Golgi generated previously was used for cis-Golgi marker (Addgene ID 97401; Park et al., 2017). The membrane-bound apoplastic marker constructs were generated by inserting a signal peptide of *At*Chitinase in front of moxVenus yellow fluorescence protein sequences (Balleza et al., 2018, Berthold et al., 2019). A vacuolar membrane marker, TONO-moxVenus, was generated by *r*TIP, vacuolar membrane protein, fusion at the N-terminal of moxVenus (Nelson et al., 2007). Plant expression constructs of GFP-*Nb*Rab13-2, apoplastic side membrane marker, and tonoplast marker were deposited to the Addgene (GFP-*Nb*Rab13-2, ID: 213473; APO-moxVenus, ID: 220490, TONO-moxVenus, ID: 220491).

### Agroinfiltration and Quantification of Cell Death Assays

*Agrobacterium tumefaciens* GV3101 strain or GV2260 containing the various constructs were cultivated overnight at 28℃ in left border (LB) medium supplemented with appropriate antibiotics. The cells were harvested through centrifugation and then resuspended in an infiltration buffer (10 mM MgCl_2_, 10 mM 2-(N-morpholino)ethanesulfonic acid (MES) [pH 5.6], and 200 μM Acetosyringone). Resuspensions were adjusted to an optical density at 600 nm (O.D. 600) of 0.1 to 0.3, and leaves of 4-week plants were agroinfiltrated for transient expression. To quantify the degree of cell death, *N. benthamiana* leaves were detached and measured using chlorophyll fluorescence, employing the default *Fv/Fm* protocol from a closed FluorCam (Photon Systems Instruments, Czech Republic). The quantification analysis was conducted using the FluorCam 7.0 software.

### Virus-Induced Gene Silencing in *N. benthamiana* and Host-Induced Gene Silencing in *P. capsici*

Virus-induced gene-silencing procedure was conducted described to the protocol described by Liu et al. (2002). *A. tumefaciens* suspensions containing pTRV1 and pTRV2 with *SGT1*, *EDS1*, *ADR1/NRG1*, and *NRC2/3/4* were mixed at a 1:1 ratio in infiltration buffer (10 mM MES, 10 mM MgCl_2_, and 200 μM Acetosyringone, pH 5.6) to a final O.D. 600 of 1.5. This mixture was infiltrated into 2 leaves of 2-week-old *N. benthamiana* plants. All plants were grown within a walk-in chamber at 24℃ under a 16-h/8-h (day/night) cycle. After 3 weeks, the upper leaves were utilized for further experiments assessing the efficiency of silencing. Host-induced gene silencing (HIGS) was conducted following the virus-induced gene-silencing procedure (Seo et al., 2022). After 3 weeks, *P. capsici* strain 40476 was inoculated onto the detached upper leaves. After 6 h, 4 leaf disks containing zoospores were collected to evaluate the silencing efficiency.

### Extraction of DNA and RNA and Gene Expression Analysis by Quantitative PCR

To assess the biomass of *P. capsici,* total genomic DNA was extracted from 4 leaf disks adjacent to the *P. capsici*-inoculated area using cetyltrimethylammonium bromide (CTAB; C2007, BIOSESANG, Korea). For gene expression analysis, total RNA was extracted from 4 leaf disks using TRIzol reagent (TR118, MRC, USA), and cDNA synthesis was conducted using Superscript II (18064014, Invitrogen, USA) following the manufacturer's instructions. Quantitative PCR and quantitative reverse-transcription PCR were performed utilizing ExcelTaq 2X Q-PCR Master Mix (SYBR, ROX; TQ1110, SMOBIO, Taiwan) with a CFX96 Touch Real-Time PCR Detection System (Bio-Rad, USA). The expression of the *Pc*Actin gene was normalized to elongation factor-1a of *N. benthamiana* (*NbEF-1a*), and the transcript levels were normalized using the internal standard (*NbEF-1a*). The primer sequences used in this study are provided in Table S4.

### Yeast 2-Hybrid Assay

Pc12 and Pc12ΔC5 were cloned into pGBKT7 vector (Takara Bio, Japan), while Rab13-2 was inserted into pGADT7 vector. The recombinant plasmids, pGBKT7 and pGADT7, were introduced into yeast strain Y2HGold and Y187, respectively. For the yeast 2-hybrid (Y2H) assay, yeast mating was performed in yeast peptone dextrose adenine (YPDA) medium at 30℃ overnight. The resulting colonies were recovered on a Synthetic Defined medium lacking tryptophan and leucine (SD/-Lue-Trp), and the interactions were validated on SD medium lacking histidine, leucine, and tryptophan (SD/-Lue-Trp-His). The medium plates were incubated at 28°C and typically photographed in 5 days. For positive and negative controls, commercial yeast constructs were used: positive control (pGBKT7-p53/pGADT7-T) and negative control (pGBKT7-p53/pGADT7-Lam), both provided by Matchmaker Gold Yeast 2-Hybrid System (630489, Clontech, USA).

### Coimmunoprecipitation, Immunoblot Assays, and Immunoprecipitation and Mass Spectrometry Analysis

*N. benthamiana* leaves infiltrated with *Agrobacterium* were sampled at 24 to 30 h post infiltration for co-IP or western blotting. Total protein was extracted using an extraction buffer (10% [v/v] glycerol, 25 mM Tris-HCl [pH 7.5], 1 mM EDTA, 150 mM NaCl, 1% [w/v] polyvinylpolypyrrolidone, and 1× protease inhibitor cocktail). The extracted proteins were immunoprecipitated with 10 μl of anti-HA magnetic beads (M180-10, MBL, Japan), anti-GFP agarose beads (D153-8, MBL, Japan), or anti-FLAG agarose beads (651502, BioLegend, USA) and incubated for 4 h or overnight at 4°C. The beads were washed 10 times with immunoprecipitation wash buffer (GTEN extraction buffer with 0.015% [v/v] Triton X-100) and resuspended in 10μl SDS loading dye. Proteins were eluted from the beads by heating at 95°C for 5 min. For western blotting, the immunoprecipitated and input proteins were separated on SDS-PAGE gels and transferred onto PVDF membranes via a Trans-Blot Turbo Transfer System (Bio-Rad, USA). After blocking the membranes with a solution of 5% skim milk prepared in PBST with 0.1% Tween 20, they were incubated with HRP anti-RFP (1:12000; M204-7, MBL, Japan), HRP anti-GFP (1:12000; AB6663, Abcam, UK), HRP anti-HA (1:12000; AB173826, Abcam, UK), or HRP anti-FLAG (1:12000; A8592, Sigma, USA) antibodies at room temperature for 1 h. The membrane was washed twice with PBST for 10 min each before ECL (1705061, Bio-Rad, USA) detection was performed according to the manufacturer’s instructions. For IP-Mass analysis, immunoprecipitated proteins were collected on SDS-PAGE gels, and the samples were analyzed using High Resolution LC/MSMS spectrometer (Q Exactive, Thermo Scientific, USA) in NICEM (National Instrumentation Center for Environmental Management, College of Agriculture and Life Sciences, Seoul National University Seoul 151-742, Korea).

### Confocal Microscopy

For confocal microscopy, 5-mm^2^ *N. benthamiana* leaf disks of the infiltrated region were observed 36 h post-*Agrobacteria* infiltration. Images were acquired with an Olympus IX83 spinning disk confocal microscope (CSW-W1 SoRA, YOKOGAWA, Japan) equipped with an ORCA-Fusion Digital CMOS camera under 60 X oil immersion objective (N.A. 1.3) by sequential detection of an average of 76 Z stacks. Images were acquired by CellSens Dimension 32 software (Olympus). The 488-nm and 561-nm laser lines (60% power) were used for EGFP and RFP, respectively. Images were processed using Fiji ImageJ (National Institutes of Health, Bethesda, Maryland, USA) from the maximum Z intensity projections of the confocal images. RFP and moxVenus/GFP were pseudo-colored magenta and green, respectively.

### Protein Structure Prediction

The structures of Pc12 and Rab13-2 were expected using AlphaFold2 in the Google Colab (https://colab.research.google.com/github/sokrypton/ColabFold/blob/main/AlphaFold2.ipynb#scrollTo=svaADwocVdwl). The predicted proteins were visualized using ChimeraX (Pettersen et al., 2021).

### Phylogenetic Analysis

In total, 149 NbRab family protein sequences were acquired from the *N. benthamiana* genome (v.1.0.1) based on the Ras domain (PF00071) and manually trimmed while considering the Rab description. In total, 57 AtRab family protein sequences were obtained from the Araport11 database of The Arabidopsis Information Resource identified by Rutherford and Moore (2002). Alignments were produced using the Multiple Sequence Comparison by Log-Expectation software in MEGAX. A phylogenetic tree was constructed using the maximum likelihood method with default parameters and 20 bootstrap replications in MEGAX (Tamura and Nei, 1993, Tamura et al., 2011).

### Statistical Analysis

Statistical analyses were performed as described in the figure legends. Prism 10 (GraphPad) was used for all statistic tests.

### Data Availability

Sequence information of protein-coding genes used in this study was obtained from the FungiDB (https://fungidb.org/), National Center for Biotechnology Information (https://www.ncbi.nlm.nih.gov/), and Sol Genomics Network (https://solgenomics.net/). The accession numbers for the sequences are as follows: Niben101Scf09596g00001.1 (Rab13-2), Niben101Scf00684g00002.1 (Rab13-3), Niben101Scf03277g02014.1 (Rab13-4), Niben101Scf05709g00001.1 (Rab13-10), Niben101Scf05032g00003.1 (Rab1), Niben101Scf16705g00001.1 (Rab8B), Niben261Chr06g1220001.1 (Rab escort protein 1 [REP1]), and Niben261Chr08g0045015.1 (prenylated Rab acceptor [PRA1]). All effector sequence information is included in Table S1. Rab13-2 (ID: 213473), an apoplastic marker (ID: 220490), and a tonoplast marker (ID: 220491) constructs are deposited in Addgene.

## RESULTS

### Pc12 Induces Cell Death Independently of the Plant Defense in Solanaceae Family

**Fig. 1:**
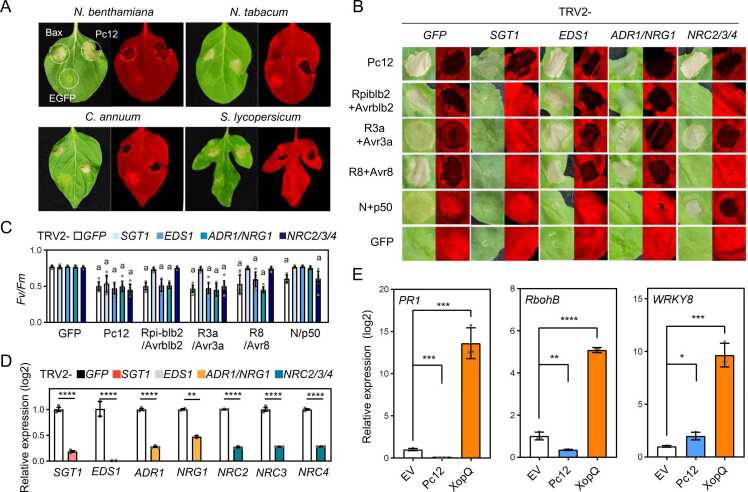
Pc12 causes cell death independent of defense response in Solanaceae family. (A) Pc12-induced cell death in *N. benthamiana, N. tobacum, C. annuum*, and *S. lycopersicum*. Bax and EGFP were used as a positive and a negative control, respectively. Leaves of 4-week-old plants were agroinfiltrated. Images were taken under white and UV light 2 days after agroinfiltration. (B) Cell death induced by Pc12 in *N. benthamiana* with silenced NLR downstream signaling genes *EDS1*, *ADR1/NRG1*, *NRC2/3/4*, and *SGT1.* Positive controls were the combinations Rpiblb2+Avrblb2, R3a+Avr3a, R8+Avr8, and N+p50, while GFP was used as a negative control. *Agrobacterium* carrying each construct was infiltrated into the leaf of 5-week-old plants silenced with the respective gene components, and images were taken at 48 hpi. (C) Cell death observed in images (B) was quantified by quantum yield (*Fv/Fm*) using a closed FluorCam system. Data represent mean ± standard deviation (SD, n = 7-10). a indicates statistically significant differences (****P < .0001). Two-way ANOVA analysis was performed followed by Dunnett’s multiple comparisons test to compare the results from GFP control with the value from silenced plants of each gene. (D) Quantitative reverse-transcription PCR analysis of transcript levels in *N. benthamiana* silencing of *SGT1, EDS1, ADR1/NRG1,* and *NRC2/3/4.* Leaf disks were sampled at 5 weeks in each component-silenced *N. benthamiana.* Asterisks indicate statistically significant differences (**P < .01; ****P < .0001) using paired 2-tailed t test. (E) Expression patterns of *PR1, RbohB*, and *WKRY8* in *N. benthamiana* expressing Pc12, XopQ, and empty vector. Total RNA was extracted at 30 hpi. Asterisks indicate statistically significant differences (*P < .1; **P < .01; ***P < .001; ****P < .0001) paired 2-tailed t test. Data are mean ± SD (3 experimental replicates).

Among the previously selected RXLR effector candidates (Seo et al., 2022), Pc12 exhibited a strong induction of cell death in *N. benthamiana*. Pc12 consistently induces cell death in other Solanaceae species when transiently expressed in *N tabacum, C annuum,* and *S. lycopersicum* (Fig. 1A), suggesting that Pc12-induced cell death might be a general response within the Solanaceae family. To determine whether the cell death induced by Pc12 is resulted from nucleotide-binding site leucine-rich repeats (NLRs)-mediated hypersensitive response (HR), we investigated Pc12-mediated cell death in *N. benthamiana* by individual silencing of NLR chaperon (*SGT1*) or NLR-downstream signaling factors, including *EDS1, ADR1/NRG1,* and *NRC2/3/4* (Botër et al., 2007, Dongus and Parker, 2021, Wu et al., 2017). Pc12-induced cell death remained unaffected by the silencing of NLR chaperon or NLR-downstream signaling components (Fig. 1B-D), suggesting that Pc12 induces cell death through mechanisms that are distinct from those typically associated with NLR-mediated effector-triggered immunity. To further validate whether the cell death induced by Pc12 is distinguished from HR cell death, we compared the transcript level of defense-related genes during Pc12 expression in contrast to the expression of a bacterial effector derived from *Xanthomonas* spp*.,* XopQ. XopQ is known to interact with the NLR protein Roq1 in *N. benthamiana*, resulting in a mild chlorotic phenotype (Schultink et al., 2017). Transcripts of defense-related genes, including *PR1, RbohB,* and *WRKY8* (Lee et al., 2023, Li et al., 2015, Moon et al., 2016, Roshan et al., 2018), exhibited significant upregulation upon transient overexpression of XopQ (Fig. 1E). In contrast, Pc12 expression did not lead to high expression of those defense-related genes, supporting the idea that Pc12-induced cell death may activate a different molecular pathway compared with the effector-triggered immunity-induced HR cell death.

### Lineage-Specific Expansion of Pc12, Contributing on *P. capsici* Virulence, and Conservation of C-Terminal Residues Critical for Cell Death Induction

**Fig. 2:**
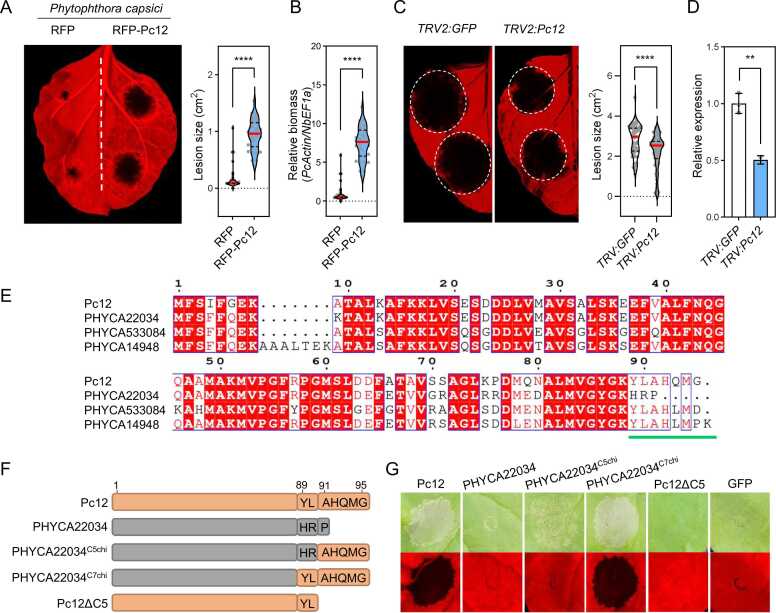
Pc12, contributing to *P. capsici* virulence, induces necrotic cell death via its C-terminus. (A) Increased *P. capsici* colonization under Pc12 expression compared with a control. RFP and RFP-Pc12 were expressed by AlcA promoter (P_alcA_) in *N. benthamiana* without ethanol. *P. capsici* was inoculated on the leaf at 24 hpi. Images were taken under UV light at 2 days after inoculation (72 hpi). Lesion sizes were measured using ImageJ. Asterisks indicate statistically significant differences (****P < .0001) using paired 2-tailed t test. Data are mean ± SD (3 experimental replicates). (B) Relative biomass of *P. capsici* in the leaf shown in (A). Leaf disks from around the inoculated area were sampled at 2 days post inoculation. Total genomic DNA was extracted and subjected to quantitative PCR analysis. The *P. capsici* biomass was quantified by the *PcActin* normalized to the *NbEF1α*. Asterisks indicate statistically significant differences (****P < 0.0001) using paired 2-tailed t test. Data are mean ± SD. (C) Suppression of *P. capsici* symptoms by silencing Pc12 using HIGS. Plants were agroinfiltrated with TRV carrying partial GFP and Pc12. At 14 days post infiltration, *P. capsici* was inoculated on the upper leaves. Images were taken at 2 days post inoculation, and the lesion size was measured using ImageJ. (D) Relative expression of Pc12 in Pc12-silenced and GFP leaves. The inoculated leaves were sampled at 6 h post inoculation and used for quantitative reverse-transcription PCR analysis. The transcript level of Pc12 was normalized to the *PcTubulin*. Asterisks indicate statistically significant differences (**P < 0.01) using paired 2-tailed t test. Data are mean ± SD. (E) Pairwise sequence alignment comparisons of Pc12 homologs from the *P. capsici* genome (LT1534). Alignments were obtained using the Multiple Sequence Comparison by Log-Expectation algorithm and visualized using ESPript 3.0 (Robert and Gouet 2014). Strictly or highly conserved residues are highlighted in red boxes or blue empty boxes, respectively. The green bar indicates the significant difference between Pc12 homologs. (F) Schematic representation of the C-terminal chimeras between Pc12 and Pc22034. (G) Cell death phenotype induced by the Pc12, Pc22034, PHYCA22034^C5chi^, PHYCA22034^C7chi^, and Pc12ΔC5 described in (F).

*Pc12* expression is elevated during the biotrophic phase of *P. capsici* compared with a biotrophic marker Haustorial membrane protein 1 (*PcHmp1*) and a necrotrophic marker Nep1-Iike protein 1 (*PcNpp1*) (Jupe et al., 2013) (Fig. S1). Although Pc12 has the canonical structure of an RXLR effector, it remains uncertain whether Pc12 contributes to the *P. capsici* virulence during colonization. To investigate the effect of Pc12 overexpression on *P. capsici* virulence, we generated the P_alcA_:RFP-Pc12 and P_alcA_:RFP constructs for leaky expression. Although the AlcA promoter (P_alcA_) is induced by ethanol treatment, leaky expression of protein of interest can occur without ethanol treatment (Lee et al., 2018). P_alcA_-driven Pc12 expression induced cell death later, at 72 h post infiltration (hpi) without ethanol treatment, compared with Pc12 expression under the cauliflower mosaic virus 35S promoter, which induced cell death earlier at 30 hpi (RFP and RFP-Pc12 were slightly detected at 0 h). The P_alcA_:RFP or P_alcA_:RFP-Pc12 constructs were agroinfiltrated into *N. benthamiana,* followed by drop inoculation of *P. capsici* on the leaves 24 hpi without ethanol treatment. A significant increase in lesion size and *P. capsici* biomass was observed at 48 h post inoculation (equivalent to 72 hpi) (Fig. 2A-B). Additionally, we used HIGS to confirm the contribution of Pc12 to *P. capsici* virulence. HIGS utilizes the host RNA interference machinery to induce targeted gene silencing in the pathogen (Cheng et al., 2015; Koch and Wassenegger, 2021; Seo et al., 2022). Knockdown of Pc12 by HIGS using tobacco rattle virus (TRV) resulted in a reduction in the pathogenicity of *P. capsici* compared with the control (Fig. 2C-D). These results suggest that Pc12 promotes the virulence of *P. capsici* in *N. benthamiana*.

To elucidate whether Pc12 functions as a single gene or as part of a gene family, we examined the copy number variation and sequence polymorphism of Pc12 homologs in *Phytophthora* spp. The sequence polymorphism of Pc12 was assessed through a BLAST search, including genome sequences of *P. capsici* (10 strains; LT1534, KPC-7, MY-1, JHAI1-7, and CPV-219/262/267/270/277/302) and 4 other *Phytophthora* spp*.* (*P. ramorum* Pr102, *P. sojae* P6497, *P. cinnamoni* CBS144.22, and *P. infestans* T30-4) retrieved from the public database (National Center for Biotechnology Information reference sequence database [RefSeq]). Our comparative genomic analysis revealed that *P. capsici* strains have a significantly higher number of Pc12 homologs in their genome compared with other *Phytophthora* spp. (Table S1, Reyes-Tena et al., 2019). To test whether Pc12 homologs could induce cell death, 3 homologs of Pc12 from *P. capsici* LT1534 and 1 homolog each from 4 other *Phytophthora* spp*.* were synthesized and transiently expressed in *N. benthamiana*. The cell death phenotype was only observed with Pc12 homologs from *P. capsici*, and not with those from other species (Fig. S2). Among the *P. capsici* homologs, only PHYCA22034 failed to elicit cell death, despite sharing 85% identity with Pc12. To identify the differences responsible for inducing cell death, sequence alignment analysis was conducted, revealing that the C-terminus may play a critical role in this process (Fig. 2E). To determine the importance of the C-terminus in Pc12-induced cell death, we generated chimeric variants by substituting the C-terminal 5 or 7 amino acids of Pc12 at the C-terminus of PHYCA22034 (PHYCA22034^C5chi^ and PHYCA22034^C7chi^) and a deletion variant lacking the last 5 amino acids (Pc12ΔC5, Fig. 2F). PHYCA22034^C5chi^ expression resulted in mild cell death, whereas PHYCA22034^C7chi^ completely restored cell death in *N. benthamiana* (Fig. 2G). Correspondingly, Pc12ΔC5 reduced the induction of cell death (Fig. 2G). These results suggest that the functional Pc12 family has expanded in a lineage-specific manner within *P. capsici*. Furthermore, the C-terminus of the Pc12 family plays a crucial role in the induction of cell death in plants.

### Pc12 Interacts With the Small GTPase Rab13-2, Localized to the Golgi Apparatus and ER, Through Its 5 C-Terminal Amino Acids

**Fig. 3:**
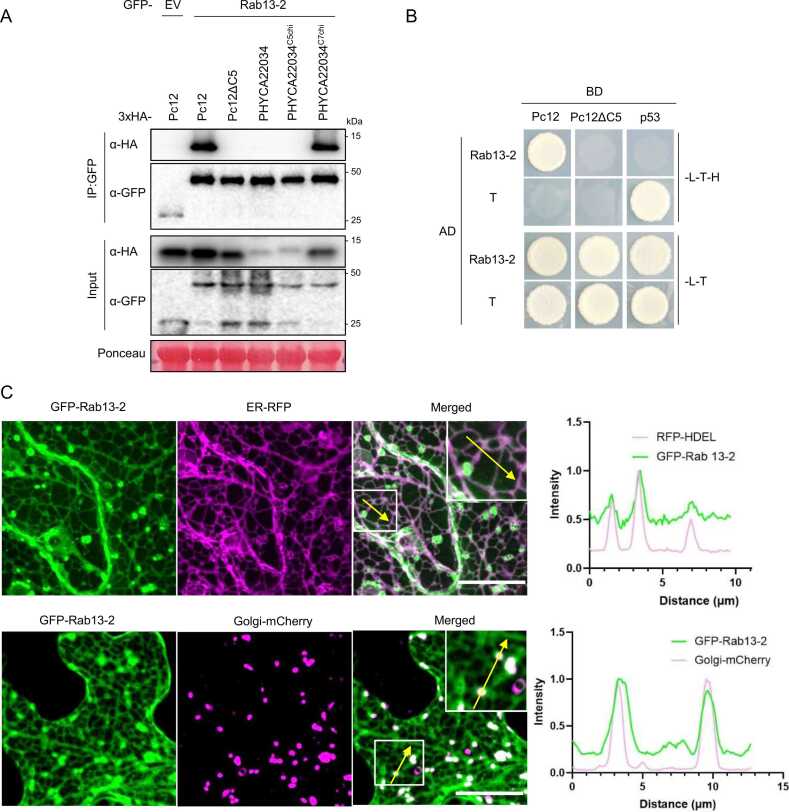
The Pc12 interacts directly with the small GTPase Rab13-2, mainly localized in the Golgi, via the C-terminus of Pc12. (A) *In planta* interaction of Rab13-2 with Pc12 and PHYCA22034 variants in Figure 2F. Pc12 and PHYCA22034 variants were transiently coexpressed with Rab13-2 in *N. benthamiana* followed by sampling at 30 hpi. (B) Physical interaction of Pc12 with Rab13-2 in vivo. Yeast cells transformed with GAL4BD-Pc12 or Pc12ΔC5 and GAL4AD-Rab13-2 were grown on synthetic media without LTH or LT for 7 days. The combination of GAL4BD-p53 and GAL4AD-T was used as a positive control. (C) Localization of GFP-Rab13-2 proteins with RFP-HDEL and cis-Golgi-mCherry, expressed in *N. benthamiana*. More than 12 images were taken from 3 independent experiments. Scale bars, 20 μm. The white box indicates the magnified region in the merged column, and the detected GFP and RFP signals along the white line are represented as intensity profiles in the graph.

To identify Pc12-interacting proteins that would be responsible for inducing cell death, we performed immunoprecipitation and mass spectrometry using Pc12 and Pc12ΔC5. In the list of proteins interacting exclusively with Pc12 but not with Pc12ΔC5, the small GTPase protein Rab13-2 was selected and identified as a genuine Pc12 target by coimmunoprecipitation (co-IP) screening, which focused on the top-10 Pc12 interactors based on the highest peptide spectrum match scores (Fig. 3A, Table S2, co-IP screening data not shown). Furthermore, direct interaction of Pc12 with Rab13-2 was also confirmed by Y2H assay (Fig. 3B). To investigate whether Pc12 homologs also interact with Rab13-2, co-IP was performed with Pc12 homologs, as shown in Figure S2. Pc12 homologs that induce cell death interacted with Rab13-2 (Fig. S4A), suggesting a potential relationship between the interaction with Rab13-2 and the induction of cell death. To further investigate whether the C-terminus of Pc12 is involved in the interaction with Rab13-2, co-IP was performed with the chimeras shown in Figure 2F. PHYCA22034^C7chi^, which induced intense cell death, interacted with Rab13-2, whereas PHYCA22034^C5chi^, which induced negligible cell death, showed no interaction with Rab13-2 (Figs. 2G and 3A). This suggests that the C-terminus of Pc12 is critical for the interaction with Rab13-2 and cell death induction.

A total of 149 genes from Rab protein family were obtained from the *N. benthamiana* genome (v.1.0.1), filtered by the Ras domain (PF00071) and Rab protein description (Table S3, Bourne et al., 1991). To predict the function of Rab13-2, a phylogenetic tree was constructed using 149 NbRab proteins and 57 AtRab proteins (Fig. S3). The 10 proteins in the Rab13 group were widely distributed throughout the phylogenetic tree and did not form a distinct cluster. Therefore, these Rab13 group proteins were named Rab13-1 to Rab13-10 in a clockwise order. To investigate the specificity of the interaction between Pc12 and Rab13-2, co-IP experiments were performed with Rab proteins, including those from the Rab13 groups as well as other Rab groups closely related to Rab13-2. In addition to Rab13-2, Pc12 was selectively bound to Rab13-3 and Rab13-4, which share 98% and 91% amino acid sequence identity with Rab13-2, respectively, but did not bind to Rab13-10 (Fig. S4B). This suggests that Pc12 preferentially interacts with specific Rab13-2 homologs.

Rab proteins are diversely localized within the plant endomembrane network, with their hypervariable C-terminal region contributing to the specificity of membrane trafficking (Bourne et al., 1991; Figueroa et al., 2001; Nielsen et at., 2008; Pylypenko et al., 2018, Stenmark, 2009). Rab13-2, the target of Pc12, was phylogenetical to AtARA3 (AtRab8A) and AtRab8B, which are localized to the ER network and Golgi apparatus (Huang et al., 2021). To specify the subcellular localization of Rab13-2, we used confocal microscopy to observe the colocalization of Rab13-2 with an ER marker, the RFP fused to ER retention signal peptides (RFP-HDEL) (Chakrabarty et al., 2007), and with a cis-Golgi marker, soybean α-1,2-mannosidase I fused to mCherry (cis-Golgi-mCherry) (Nelson et al., 2007, Park et al., 2017). GFP-Rab13-2 signals mainly colocalized with cis-Golgi-mCherry signals and RFP-HDEL (Fig. 3C), suggesting that Pc12 interactor, Rab13-2, may function for the vesicle trafficking at the ER-Golgi interface.

### Pc12 Interferes With Rab13-2 Vesicle Trafficking at the ER-Golgi Interface and Increases the Affinity of REP1 for Rab13-2

**Fig. 4:**
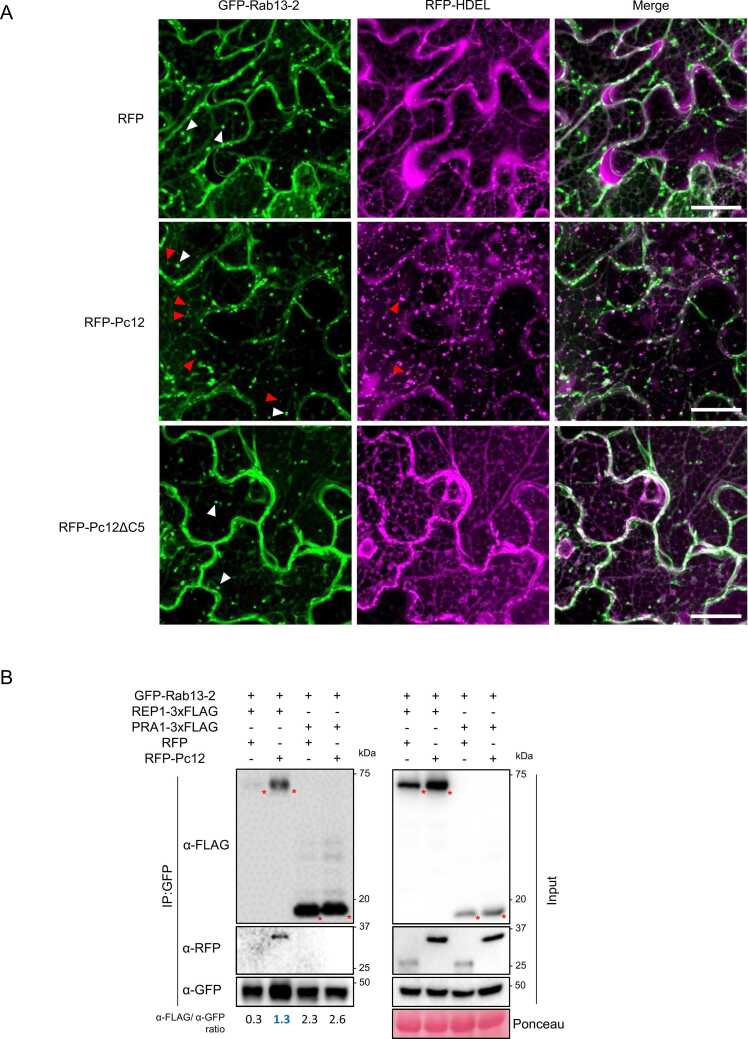
Pc12 relocalizes to the ER and Rab13-2 and increases the affinity of REP1 for Rab13-2. (A) The expression of RFP, RFP-Pc12, and RFP-Pc12ΔC5 was induced by 1% ethanol in transgenic *N. benthamiana* expressing RFP-HDEL and GFP-Rab13-2. More than 60 z-images with a step size of 0.3 μm were acquired using a spinning disc confocal microscope. Images were superimposed using a maximum z-projection function in ImageJ. More than 12 images were taken from 3 independent experiments. Scale bars, 20 μm. The white arrowhead indicates the globular shape represented by GFP-Rab13-2, and the red arrowhead highlights puncta-like aggregation. (B) co-IP assays showing that Pc12 increases the binding affinity between Rab13-2 and REP1. Plants transiently expressing GFP-Rab13-2, REP1- or PRA1-3xFLAG, and RFP or RFP-Pc12 were sampled at 12 h after a 1% ethanol treatment at 24 hpi. Total protein extracts were subjected to co-IP using anti-GFP agarose beads. The precipitated proteins were immunoblotted. The amount of binding was calculated as the ratio of anti-FLAG and anti-GFP of the IP using imageJ. Red asterisks indicate the expected band sizes, and blue letters highlight the increased band signal with α-FLAG/α-GFP = 1.3.

**Fig. 5:**
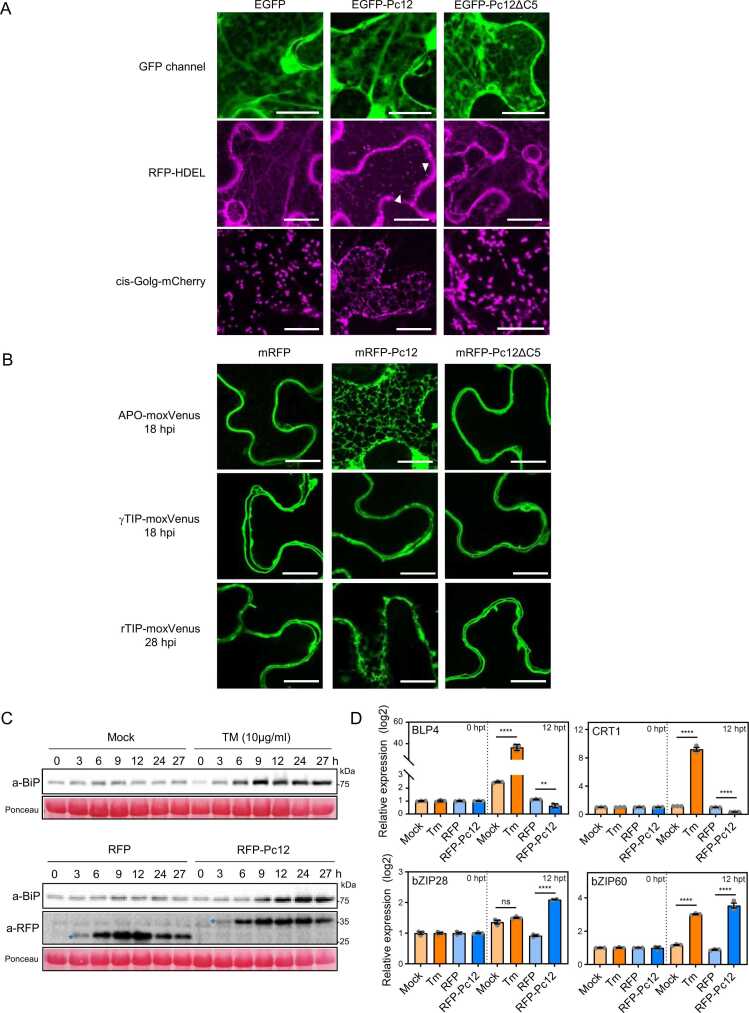
Pc12 disrupts the ER-Golgi interface, affecting the secretory pathway and causing ER stress distinct from Tm-dependent stress. (A) Effects of EGFP-Pc12 on the localization of RFP-HDEL and mCherry-cis-Golgi marker. GFP, GFP-Pc12, and GFP-Pc12ΔC5 were transiently coexpressed with RFP-HDEL and mCherry-cis-Golgi marker in *N. benthamiana*. Proteins were imaged using a spinning disc confocal microscope. More than 60 z-images in 0.3-μm steps were superimposed using a maximum z-projection function in ImageJ. More than 12 images were obtained from 3 independent experiments. Scale bars, 20 μm. The white arrowhead highlights puncta-like aggregation. (B) ApoSP-moxVenus remained in the ER in the presence of Pc12, and not-affected γ-TIP-moxVenus was localized in the tonoplast. RFP-Pc12, RFP-Pc12ΔC5, and RFP were induced by 1% ethanol. Z-images for sections thicker than 3 μm in 0.75-μm step size were acquired using a laser scanning confocal microscope. Images of the corresponding sections have been processed to improve the brightness for clarity. This processing does not change the conclusions drawn from the images. moxVenus was pseudocolored to green. More than 12 images were taken from 3 independent experiments. Scale bars, 20 μm. (C) Accumulation of the ER stress marker protein (BiP) in response to Pc12 expression. Plants were treated with mock and Tm (10 μg/ml) and sampled over time. Plants expressing RFP and RFP-Pc12 after 1% ethanol treatment were sampled over time. (D) Transcript levels of UPR-related transcription factors and ER chaperon genes upon expression of Pc12. In (C), total RNA was extracted at 0 and 12 h after Tm or 1% ethanol treatment. UPR-related genes, ER chaperon genes (*BLP4* and *CRT1*), and transcription factor genes (*bZIP28* and *bZIP60*) were normalized with *NbEF-1a* in quantitative reverse-transcription PCR. Asterisks indicate statistically significant differences (****P < .0001) using paired 2-tailed t test. Data are mean ± SD.

To gain insight into the localization of Pc12 in host cells, we transiently expressed EGFP-Pc12 and EGFP-Pc12ΔC5 (without the signal peptide and RXLR-EER motifs). The localization of EGFP-Pc12 and EGFP-Pc12ΔC5 was similar to that of free EGFP, with cytoplasmic localization. This observation suggests that Pc12 is distributed throughout the plant cell, rather than being confined to specific compartments. To investigate the effect of Pc12 on Rab13-2, we generated transgenic *N. benthamiana* plants expressing GFP-Rab13-2 together with the RFP-HDEL (Fig. 4A) and agroinfiltrated with the P_alcA_:RFP-Pc12 construct. To avoid spectral overlap, Pc12 should be fused with other fluorescent proteins other than GFP and RFP. However, the signals of EGFP-Pc12 and EGFP-Pc12ΔC5 were nearly identical to those of the negative control, EGFP alone (Fig. 5A). This suggests that these proteins are unlikely to affect RFP-HDEL signals observed under the confocal microscope unless they directly interact with or impact on RFP-HDEL proteins. Furthermore, Rab13-2 localizes to the ER and Golgi apparatus (Fig. 3C), suggesting Rab13-2 may play a role in vesicle trafficking between these 2 organelles. This implies that observing the localization of both Rab13-2 and the ER together is essential to understand potential functional interactions in the presence of Pc12. When RFP and RFP-Pc12ΔC5 were expressed following 1% ethanol treatment on day 1 after agroinfiltration, the RFP channel showed a reticular morphology, similar to that observed with only RFP-HDEL in Figure 3C (Fig. 4A). Interestingly, under RFP-Pc12 expression, an aggregation phenotype was observed in the RFP channel at 18 h post ethanol treatment, where RFP-Pc12 was fully expressed and no cell death was detected under the microscope (Fig. 4A, RFP-Pc12 in Fig. 5C, and γ-TIP-moxVenus at 18 hpi in Fig. 5B). In addition, the typical globular morphology expected for the Golgi, where Rab13-2 is mainly localized, was rarely observed in the GFP channel. Instead, smaller aggregates, different from normal Golgi structures, were frequently observed (Fig. 4A). This observation suggests that Pc12 interferes with the canonical localization of Rab13-2 and RFP-HDEL.

Rab proteins are required to interact with or recruit other proteins to function in vesicle budding, trafficking, and fusion processes (Nielsen et al., 2008, Pylypenko et al., 2018, Stenmark, 2009). To identify interactors of Rab13-2 that are influenced by Pc12 expression, we blasted Rab13-2 sequence on the STRING database (Table S4). We cloned 8 genes from *N. benthamiana* cDNA and performed co-IP with these candidates and Rab13-2 (Fig. S5). Six candidates were detected via western blotting of the input, whereas RI3 (ethylene-responsive transcription factor RAP2-3) and RI8 (phosphatidylinositol 4-phosphate 5-kinase 2-like) were not, possibly due to instability in protein synthesis or degradation process. Among 6 interactor candidates, the REP1 and the PRA1 were identified as interacting with Rab13-2 through co-IP screening (Fig. S5). REP1 functions as a chaperon for the prenylation of Rab proteins escorts Rab proteins to their target membranes, and self-recycles throughout these processes (Guo et al., 2008, Nielsen, 2020). PRA1 acts as a receptor for prenylated Rab proteins, promoting their binding to membranes (Figueroa et al., 2001). To assess the effect of Pc12 on these interactions, we performed a co-IP experiment. Under the microscope, Pc12 induced the aggregation of RFP-HDEL, an ER-resident protein (Fig. 4A), suggesting that Pc12 expression may influence the accumulation of transiently overexpressed proteins. Based on this observation, we designed a protein expression strategy where REP1, PRA1, and Rab13-2 were expressed in *N. benthamiana* using the 35S promoter, while P_alcA_:RFP-Pc12 was induced by 1% ethanol treatment on day 1 post agroinfiltration. REP1 and PRA1 bound to Rab13-2, whereas Pc12 interacted with Rab13-2 only in the presence of REP1 but not in the presence of PRA1 (Fig. 4B). This suggests that Pc12 cannot associate with the Rab13-2/PRA1 complex. Interestingly, the interaction between REP1 and Rab13-2 was enhanced in the presence of Pc12 (blue asterisk, α-FLAG/α-GFP = 1.3, Fig. 4B). This suggests that Pc12 may stabilize the REP1/Rab13-2 complex, thereby affecting the recycling of REP1 and Rab13-2 for vesicle trafficking at the ER-Golgi interface.

### Pc12 Induces a Distinct ER Stress by Disrupting the ER-Golgi Interface, Affecting the Secretory Pathway

Although the RFP channel showed RFP-HDEL and RFP-Pc12 simultaneously in Fig. 4A, aggregation was observed only under Pc12 expression, unlike the reticular morphology of RFP-HDEL. To further investigate the structural changes in ER and Golgi in response to Pc12, EGFP-Pc12 was coexpressed with RFP-HDEL or cis-Golgi-mCherry in *N. benthamiana*. Under EGFP and EGFP-Pc12ΔC5 expression, RFP-HDEL typically showed a reticular ER morphology, and cis-Golgi-mCherry showed a globular Golgi structure under the confocal microscope (Fig. 5A). Interestingly, EGFP-Pc12 expression altered morphologies of RFP-HDEL to show a peculiar punctate signal and of cis-Golgi-mCherry to be reminiscent of the ER tubules (Fig. 5A). In the normal condition, newly synthesized proteins containing HDEL are initially synthesized in the ER lumen, and then transported to the Golgi apparatus where HDEL receptors bring HDEL-containing proteins back to the ER via vesicles (Alvim et al., 2023). The mislocalization of RFP-HDEL and cis-Golgi-mCherry in the presence of Pc12 suggests that Pc12 interferes with the serial recycling of cargo through vesicle trafficking at the ER-Golgi interface. To investigate whether Pc12-mediated disruption of trafficking across the ER-Golgi interface affects protein secretion, we monitored the localization of 2 secretion markers, an apoplastic membrane-targeted moxVenus (ApoSP-moxVenus, Balleza et al., 2018) and a tonoplast marker (γ-TIP-moxVenus, Nelson et al., 2007). ApoSP-moxVenus is secreted from the ER to the apoplastic region through mature processing and delivery by the Golgi apparatus (Balleza et al., 2018). γ-TIP-moxVenus moves from the ER to the vacuole, bypassing the Golgi as a route of ER-vacuole trafficking (Rojas-Pierce, 2013). The typical apoplastic localization of ApoSP-moxVenus was observed under RFP and RFP-Pc12ΔC5 expression. However, in the presence of RFP-Pc12, ApoSP-moxVenus was completely retained within the ER at 18 h after 1% ethanol treatment (Fig. 5B). In contrast to ApoSP-moxVenus, the localization of γ-TIP-moxVenus 18 h after mRFP-Pc12 induction was similar with that observed with mRFP and mRFP-Pc12ΔC5 (Fig. 5B). Over time, at 28 and 32 h post ethanol treatment, γ-TIP-moxVenus exhibited a net-like morphology within the dead cells, suggesting that the vacuoles may have undergone degradation in the dead cells (Huysmans et al., 2017; Fig. S6). At this point, we hypothesized that Pc12 alternation of Rab13-2 dynamics may cause a secretion traffic jam at the ER-Golgi interface, leading to ER stress-induced necrotic cell death.

Pc12 expression induces changes similar to those reported by Qiang et al., (2012, Fig. 5B). We compared the responses triggered by Pc12 and tunicamycin (Tm), a well-known ER stress inducer. These responses include the accumulation of ER chaperon-binding immunoglobulin protein (BiP) and the transcription of UPR genes (Adams et al., 2019, Angelos et al., 2017, Cao and Kaufman, 2012, Yang et al., 2014). Ethanol-induced RFP-Pc12 expression and Tm treatment resulted in the gradual accumulation of BiP over time (Fig. 5C). To evaluate the expression of UPR-related genes, leaf samples treated with Tm and 1% ethanol to induce expression of RFP or RFP-Pc12 were collected at 0 and 12 h after treatment. Tunicamycin treatment led to upregulated transcripts for both ER chaperons (BiP-like protein *BLP4* and calreticulin *CRT1*) and ER stress-related transcription factors (*bZIP28* and *bZIP60*) (Adams et al., 2019, Yang et al., 2014). However, Pc12 expression did not lead to upregulate the ER chaperon transcripts but did increase the expression of *bZIP28* and *bZIP60* (Fig. 5D). These findings suggest that Pc12 induces an ER stress, distinct from that induced by Tm.

### A Specific Residue of Rab13-2 Interacting With the C-Terminus of Pc12 Is Crucial for Pc12 Targeting

**Fig. 6:**
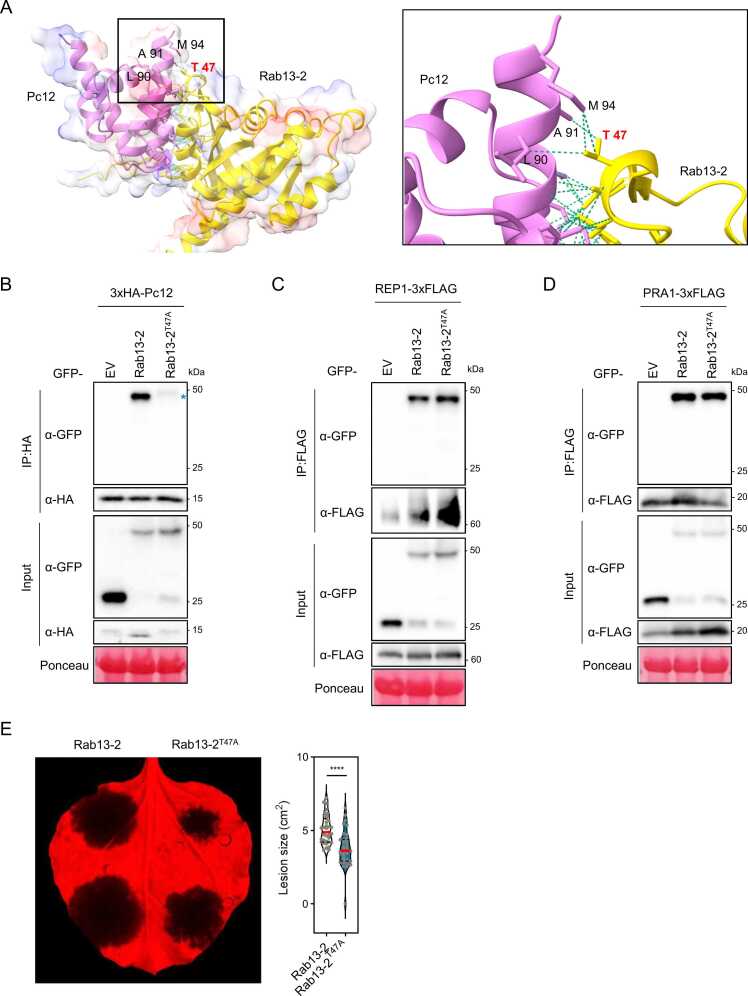
A mutation in a key Rab13-2 residue weakens Pc12 binding without affecting REP1 and PRA1 binding, compromising *P. capsici* virulence. (A) The predicted structures of Pc12 and Rab13-2 interaction by AlphaFold2. The right black box was magnified from the black box of the left docking model. The green lines indicate the intracellular hydrogen binds formed between Pc12 and Rab13-2. (B-D) *In planta*, co-IP assay with Rab13-2, Rab13-2^T47A^, and Pc12 (B), REP1 (C), or PRA1 (D). The leaves were sampled at 48 hpi and total protein extracts were precipitated with anti-HA magnetic beads or anti-FLAG agarose. Precipitated and total proteins were detected by western blotting. The blue asterisk highlights the reduced binding affinity of REP for Rab13-2^T47A^. (E) *P. capsici* inoculation on the leaves expressing GFP-Rab13-2 and GFP-Rab13-2^T47A^. *Agrobacterium* carrying GFP-Rab13-2 and GFP-Rab13-2^T47A^ was infiltrated into *N. benthamiana,* followed by *P. capsici* inoculation at 24 hpi. Images were taken at 36 h post inoculation (60 hpi), and the lesion size was measured using ImageJ (N = 31-32, 3 replicates). Asterisks indicate statistically significant differences (****P < .0001) using paired 2-tailed t test. Data are mean ± SD.

To better characterize the biochemical properties of the Pc12 and Rab13-2 interaction, we utilized the AlphaFold2 program (Jumper et al., 2021) to identify a critical residue on Rab13-2 that interacts with the 5 C-terminal amino acids (AHQMG) of Pc12, which are essential for the interaction. Rab13-2 was predicted to have a globular structure, and Pc12 displayed a stack of 3 parallel α-helices connected by a short helical linker (Fig. 6A). A docking model proposed that the 3 parallel α-helices of Pc12 interacted with one side of the globular region of Rab13-2. Notably, Leu90, Ala91, and Met94 of Pc12 were predicted to engage with Thr47 of Rab13-2 (Fig. 6A). To determine whether Thr47 of Rab13-2 is necessary for the interaction between Pc12 and Rab13-2, a point mutation substituting threonine for alanine (Rab13-2^T47A^) was generated for co-IP analysis and expressed by 35S promoter. Rab13-2^T47A^ showed a reduced interaction with Pc12 (Fig. 6B). To test whether the point mutation in Rab13-2 affects interactions with REP1 and PRA1, co-IP was performed. The results showed that REP1 and PRA1 bind to Rab13-2^T47A^, suggesting that the point mutation on Thr47 of Rab13-2, essential for the interaction with the C-terminal amino acids of Pc12, does not affect the binding of REP1 and PRA1 (Fig. 6C-D). To evaluate the impact of Rab13-2^T47A^ on *P. capsici* pathogenicity, Rab13-2 and Rab13-2^T47A^ were transiently expressed in *N. benthamiana*, followed by inoculation with *P. capsici* at 24 hpi*.* At 48 h post inoculation (72 hpi), the lesion size was significantly reduced in the leaf expressed with Rab13-2^T47A^ compared to those expressing Rab13-2 (Fig. 6E). Considering the intrinsic role of the endogenous Rab13-2, this result suggests that bypassing the Pc12 targeting could reduce the susceptibility of *N. benthamiana* to *P. capsici*.

## DISCUSSION

### Pc12 Serves as a Transition Effector, Orchestrating the Transition From the Biotrophic to the Necrotrophic Life Cycle of *P. capsici*

*P. capsici* is classified as a hemibiotrophic pathogen, characterized by a biphasic life cycle. It initiates infection through a biotrophic phase, establishing itself within living host cells. This is followed by a necrotrophic phase, where the pathogen extracts nutrients from dead host cells, leading to sporulation that facilitates dissemination (Kamoun et al., 2015, Parada-Rojas et al., 2021, Quesada-Ocampo et al., 2023). Although researchers have elucidated the roles of RXLR effectors in enhancing susceptibility by interfering with host cell physiology or triggering defense responses through interactions with R proteins during the biotrophic phase, our understanding of how RXLR effectors induce necrosis in host cells remains limited. Recently, a case of necrosis in which a *P. infestans* RXLR effector was reported to induce nucleolar inflammation and impede pre-rRNA 25S processing, leading to necrosis through disruption of protein translation (Lee et al., 2023b). This study revealed that the RXLR effector Pc12 functions as a virulence factor, inducing ER stress and necrosis by hindering functions of Rab13-2 in vesicle trafficking. Furthermore, evasion of Pc12 targeting by Rab13-2^T47A^ led to a reduction in lesion size, highlighting that Pc12-induced necrosis with binding Rab13-2 contributes to the pathogenicity. Pc12 expression was highly upregulated at 6 h after inoculation, while the transcripts of NPP1, necrosis-inducing peptides, gradually increased at 12 h post inoculation (Fig. S1). This raises the question of why the transcription of Pc12 increased early in infection, despite its role in inducing necrosis. In the experiment using the ethanol-inducible promoter, although leaky expression was observed at 0 h, significant Pc12 protein accumulation became detectable at 3 h after 1% ethanol treatment, as shown by immune blots (Fig. 6C). Cell death was observed prior to 24 h after 1% ethanol treatment, as evidenced by microscopy (Fig. S6). Generating Pc12-knockout *P. capsici* transformants or *Phytophthora* spp. transformants overexpressing Pc12 would enable a more comprehensive assessment of the impact of Pc12 on *P. capsici* pathogenicity. However, generating *P. capsici* transformants with Pc12 knockouts is challenging due to the presence of multiple Pc12 homologs in *P. capsici* genome. Additionally, producing *Phytophthora* spp. transformants that overexpress Pc12 remain technically difficult due to current limitations in transformation efficiency. However, despite these limitations, given the time delay between gene transcription and necrosis induction, it could be inferred that Pc12-induced necrosis serves as an initiator for the transition into the necrotrophic phase.

### Pc12 Has Emerged After the Evolutionary Divergence of *P. capsici* to Inhibit Host Defense

Within the genus *Phytophthora*, only *P. capsici* possesses multiple copies of at least 8 and up to 29 Pc12 homologs with the potential to induce necrosis. In contrast, Pc12 homologs from *P. ramorum*, *P. cinnamoni*, and *P. sojae* failed to induce cell death (Table S1, Fig. S2). *P. infestans* lacks a Pc12 homolog, despite its close evolutionary relationship with *P. capsici* (Lamour et al., 2012, Sharma et al., 2015). This suggests that the Pc12 family may have evolved after the divergence of *P. capsici* and may have undergone lineage-specific expansion within the species. Proteins involved in vesicle trafficking are commonly targeted by effectors from various pathogens. For instance, RabE1c, which is crucial in the secretion pathway, is targeted by *Pseudomonas syringae* AvrPto and *P. infestans* PexRD12/31 family (Petre et al., 2021, Speth et al., 2009). Similarly, Rab8A is targeted by *P. infestans* PexRD54 and *Agrobacterium tumefaciens* VirB2 (Huang et al., 2021, Pandey et al., 2021). These interactions suggest that such effectors manipulate vesicle trafficking of defense proteins to enhance host susceptibility, facilitating pathogen establishment. This research demonstrates that Pc12 interferes with Rab13-2-mediated vesicle trafficking at the ER-Golgi interface, leading to ER stress and inhibiting the secretion pathway. These findings suggest that neofunctionalized Pc12, by effectively disrupting Rab13-2-mediated vesicle trafficking and contributing to *P. capsici* virulence, might also potentially stimulate the expansion of Pc12 homologs within the genome.

### Rab13-2-Mediated Vesicle Trafficking at the ER-Golgi Interface and Pc12 Causes ER Stress and Necrotic Cell Death

Unlike 57 Rab proteins in *Arabidopsis* (Rutherford and Moore, 2002), *N. benthamiana* has 149 Rab superfamily genes. Rab13-2 is closely positioned with AtARA3 (AtRab8A) and AtRab8B in the phylogenetic tree (Fig. S3). In mammals and yeast, Rab8 is known to facilitate polarized vesicle transport from the trans-Golgi network to the plasma membrane (Huang et al., 2021) However, in *Arabidopsis*, AtRabE1 (isoform A-E) serves this function, and AtRabE1 is distantly related to Rab13-2 (Speth et al., 2009, Zheng et al., 2005; Fig. S3). AtRab8 (isoforms A, B, and D) is localized at ER networks and the Golgi apparatus in *Arabidopsis*, where it co-localizes with Reticulon-like B proteins involved in ER tubular structure formations for intracellular trafficking (Huang et al., 2021). This study shows that Rab13-2 of *N. benthamiana* is localized at Golgi apparatus and ER network (Fig. 3C), suggesting that Rab8 proteins involved in endomembrane trafficking are distributed differently depending on the organisms, with plant cells specifically featuring localization to the ER network and Golgi apparatus.

**Fig. 7:**
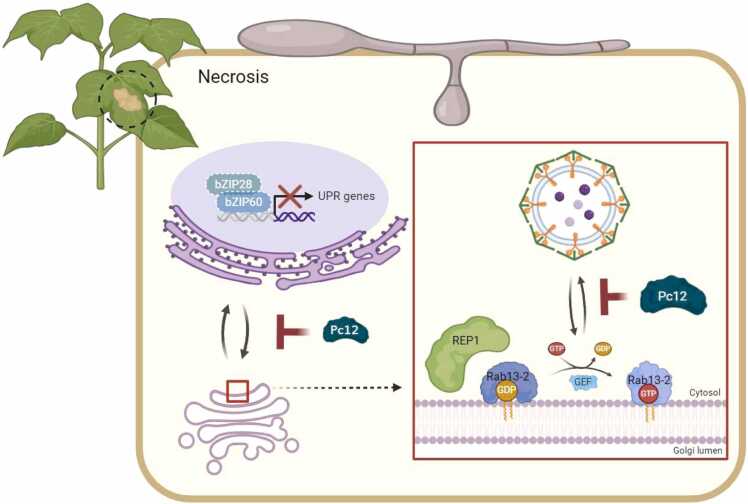
Summary model of Pc12 inhibition of Rab13-2-mediated vesicle trafficking at the ER-Golgi interface. Pc12, secreted by *P. capsici*, stabilizes the complex of Rab13-2 and REP1, preventing vesicle trafficking at the ER-Golgi interface and ultimately causing severe ER stress leading to necrosis. This figure was created using BioRender.com.

Proteins synthesized in ER are transported to subcellular compartments through Golgi apparatus, while some proteins, such as tonoplast protein and soluble proteins, use the Golgi-independent pathways to reach the vacuole (De Marchis et al., 2013). This study showed that, in the presence of Pc12, ApoSP-moxVenus was confined to the ER, whereas γ-TIP-moxVenus remained localized at the tonoplast 18 h after Pc12 expression induced by 1% ethanol within alive cells (Fig. 5B). The altered signals of γ-TIP-moxVenus were observed at 28 and 36 h after 1% ethanol with Pc12-induced cell death (Fig. S6). These results suggest that Pc12 specifically disrupts vesicle trafficking on the ER-Golgi interface in the conventional secretion pathway but not the Golgi bypassing pathway (Fig. 7). Pc12 definitively disrupts the ER-Golgi interface; however, additional detailed investigations are required to ascertain whether Rab13-2 is implicated in this disruption.

Mutualistic fungus *Piriformospora indica* induces ER stress-triggered cell death upon colonizing *Arabidopsis* roots (Qiang et al., 2012). Pc12 induces ER stress characterized by BiP accumulation and bZIP28/60 upregulation but does not lead to upregulation of the bZIP28/60 downstream targets BLP4 and CRT1, unlike Tm (Fig. 5). This suggests that Pc12-induced ER stress does not trigger the nuclear translocation of bZIP28/60, leading to prolonged unresolved ER stress and subsequent necrotic cell death. While severe ER stress is known to induce necrosis, the factors connecting severe ER stress and necrosis remain unclear. Therefore, Pc12 may serve as a valuable model for studying the relationship between severe ER stress and necrotic cell death.

### Pc12 Increases the Binding Affinity of REP1 for Rab13-2, and Changing the Targeting Site of Rab13-2 Can Prevent Pc12 Targeting

REP1 self-recycles by binding to Rab proteins during their prenylation and subsequently releasing Rab proteins to target membranes (Guo et al., 2008, Nielsen, 2020). This process suggests that the binding affinity of REP1 for Rab proteins may be relatively low, enabling efficient recycling of both REP1 and Rab proteins. Pc12 significantly enhances the binding affinity of REP1 for Rab13-2 (Fig. 4B). This suggests that Pc12 may stabilize the REP1/Rab13-2 complex, leading to inhibition of REP1 recycling and preventing Rab13-2 from anchoring at the destination membrane (Fig. 7). To investigate this scenario, the equilibrium dissociation constant of the REP1/Rab13-2-Pc12 complex should be determined by using isothermal titration calorimetry experiments. However, due to the allosteric conformational changes of REP1 and Rab proteins in response to different binding partners, isothermal titration calorimetry may present challenges in elucidating their interactions (Anant et al., 1998, Rak et al., 2004).

The Rab13-2^T47A^ mutant, which alters the Thr47 interacting with the C-terminal residues of Pc12, exhibits weakened binding to Pc12. However, the binding affinity of Rab13-2^T47A^ for REP1 and PRA1, which are interactors of Rab13-2, remains unaffected (Fig. 6A-D). This suggests that Rab13-2^T47A^ may reduce the impact of Pc12 on the REP1/Rab13-2 complex. The underlying molecular mechanism of host resistance is believed to arise from (1) preexisting barriers preventing pathogen colonization, (2) the detection of pathogen effector proteins triggering robust immunity, and (3) the incapacity of pathogen effectors to suppress immunity triggered by molecular patterns (McLellan et al., 2022). Expression of Rab13-2^T47A^ reduced *P. capsici* colonization in *N. benthamiana*, suggesting that avoiding Pc12 targeting may contribute to the resistance of *N. benthamiana* against *P. capsici.*

### Limitations of This Research

Since Pc12 disrupts Rab13-2 functioning in the vesicle trafficking at the ER and Golgi apparatus, affecting the cell viability, silencing of Rab13-2 and homologs (Rab13-3 and Rab13-4) resulted in a lethal phenotype. Notably, the expression of Rab13-2^T47A^ in WT *N. benthamiana* prevented Pc12 targeting and reduced *P. capsici* colonization (Fig. 6B-E). Therefore, completely replacing endogenous Rab13-2 and its homologs with Rab13-2^T47A^ (not performed in this study) by generating stable transformants could provide further insights by mitigating the effects of Pc12 on Rab13-2. This approach would enable direct validation of involvement of Rab13-2 in necrosis and provide a model for resistance mechanisms against pathogens.

## AUTHOR CONTRIBUTIONS

**Jesse Kaleku:** Visualization. **Haeun Kim:** Resources, formal analysis. **Minji Kang:** Methodology. **Hui Jeong Kang:** Resources. **Jongchan Woo:** Writing—review and editing, methodology, and funding acquisition. **Hongshi Jin:** Methodology. **Seungmee Jung:** Methodology. **Cécile Segonzac:** Resources. **Eunsook Park:** Writing—review and editing, supervision, project administration, funding acquisition, and conceptualization. **Doil Choi:** Writing—review and editing, supervision, project administration, funding acquisition, and conceptualization. **Jihyun Kim:** Writing—review and editing, writing—original draft, visualization, validation, methodology, investigation, and data curation.

## DECLARATION OF COMPETING INTERESTS

The authors declare that they have no known competing financial interests or personal relationships that could have appeared to influence the work reported in this paper.

## ORCID

Jihyun Kim: https://orcid.org/0000-0003-0195-4319.

Jesse Kaleku: https://orcid.org/0000-0002-7089-1609.

Jongchan Woo: https://orcid.org/0000-0002-2994-6444.

Hongshi Jin: https://orcid.org/0000-0003-3283-1012.

Hui Jeong Kang: https://orcid.org/0000-0002-1347-8965.

Minji Kang: https://orcid.org/0000-0001-8118-3184.

Haeun Kim: https://orcid.org/0000-0002-6171-0650.

Seungmee Jung: https://orcid.org/0000-0003-2660-7663.

Cecile Segonzac: https://orcid.org/0000-0002-5537-7556.

Eunsook Park: https://orcid.org/0000-0003-2984-3039.

Doil Choi: https://orcid.org/0000-0002-4366-3627.

## Footnotes

Appendix A Supplementary data associated with this article can be found in the online version at doi:10.1016/j.mocell.2024.100158.
