# Supplementary material for: An RXLR effector disrupts vesicle trafficking at ER-Golgi interface for Phytophthora capsici pathogenicity
Source: Mol Cells. 2024 Nov 20;47(12):100158. doi: 10.1016/j.mocell.2024.100158 (PMC11683230; doi:10.1016/j.mocell.2024.100158)
Supplement: Supplementary file 1 — Supplementary material [file mmc1.pdf]

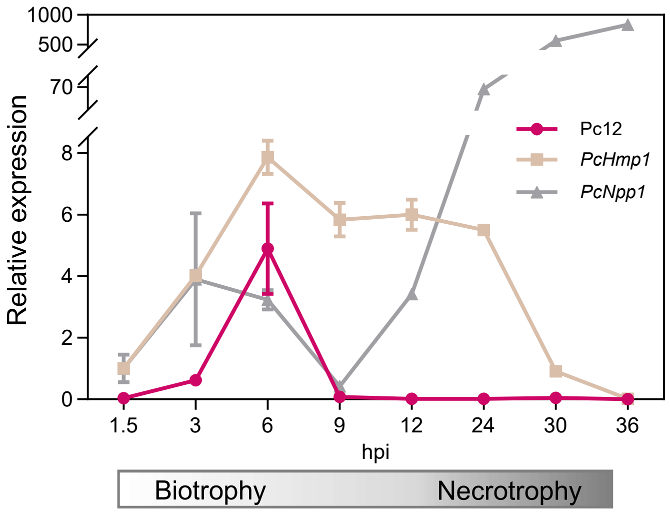

Figure S1. Expression pattern of Pc12 during the infection phases of *P. capsici*. *PcHmp1* and *PcNpp1* are used as markers for the biotrophic and necrotrophic phases, respectively. Leaf disks were sampled at regular time intervals. Each gene is normalized to *PcTubulin*.

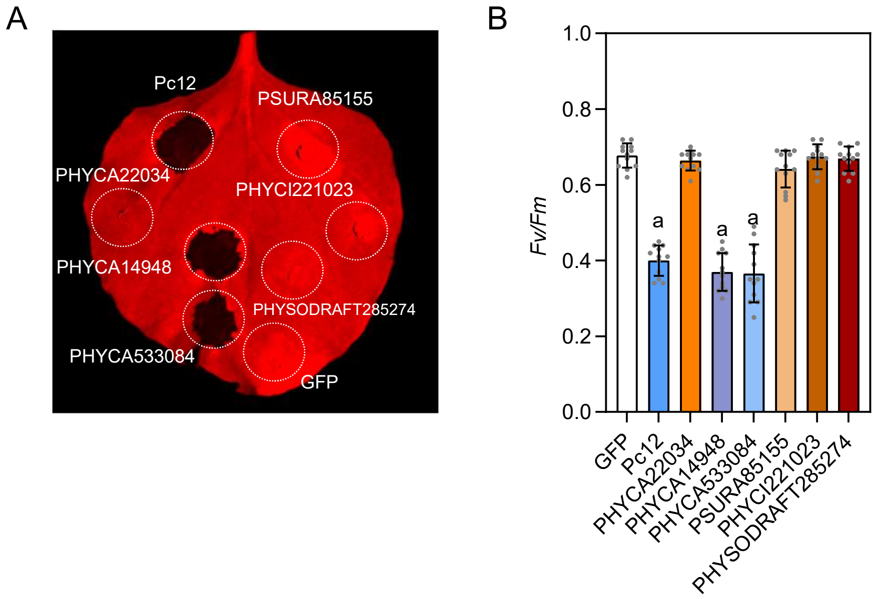

Figure S2. Pc12 homologs from *P. capsici* induce cell death, except for PHYCA22034.
(A) Cell death analysis of Pc12 homologs from *P. capsici, P. ramorum, P. cinnamoni,* and *P. sojae.* Pc12 homologs were expressed in 4-week-old *N. benthamiana* and images were taken 2 days after agroinfiltration.
(B) Cell death in images (A) was quantified by quantum yield (*Fv/Fm*) using a closed FluorCam system. Data are mean ± SD (n = 9-12). a indicates statistically significant differences (****, $P < 0.0001$) using unpaired two-tailed t test. Data are mean ± SD.

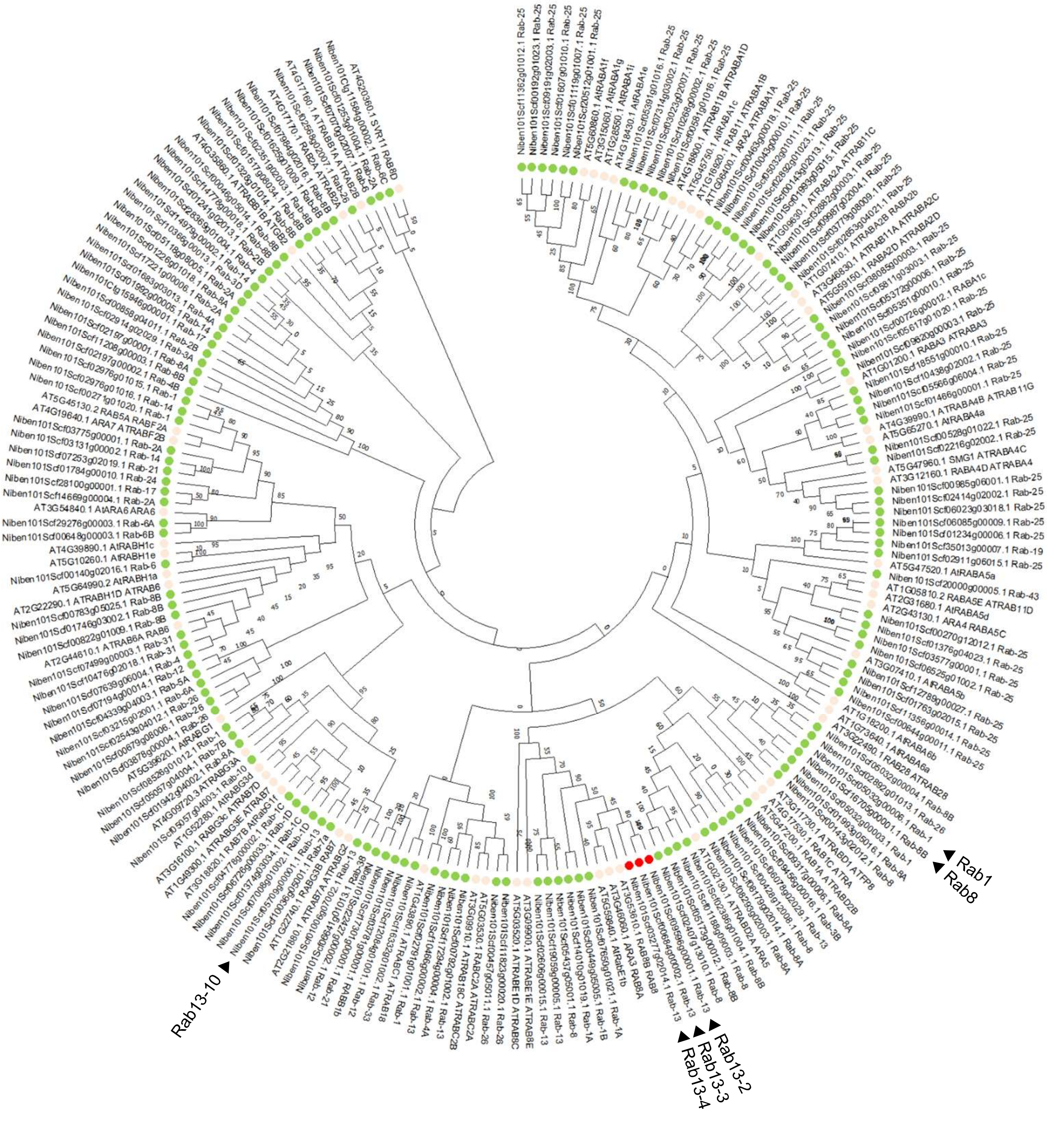

Figure S3. Phylogenetic tree of the Rab family in *N. benthamiana* and *Arabidopsis*
The black triangle represents the Rab proteins used in the co-IP shown in Figure S4. The red circle indicates Pc12 targets in Figure S4. The green circle represents Rab genes in *N. benthamiana*, and the ivory circle denotes Rab genes in *A. thaliana*.

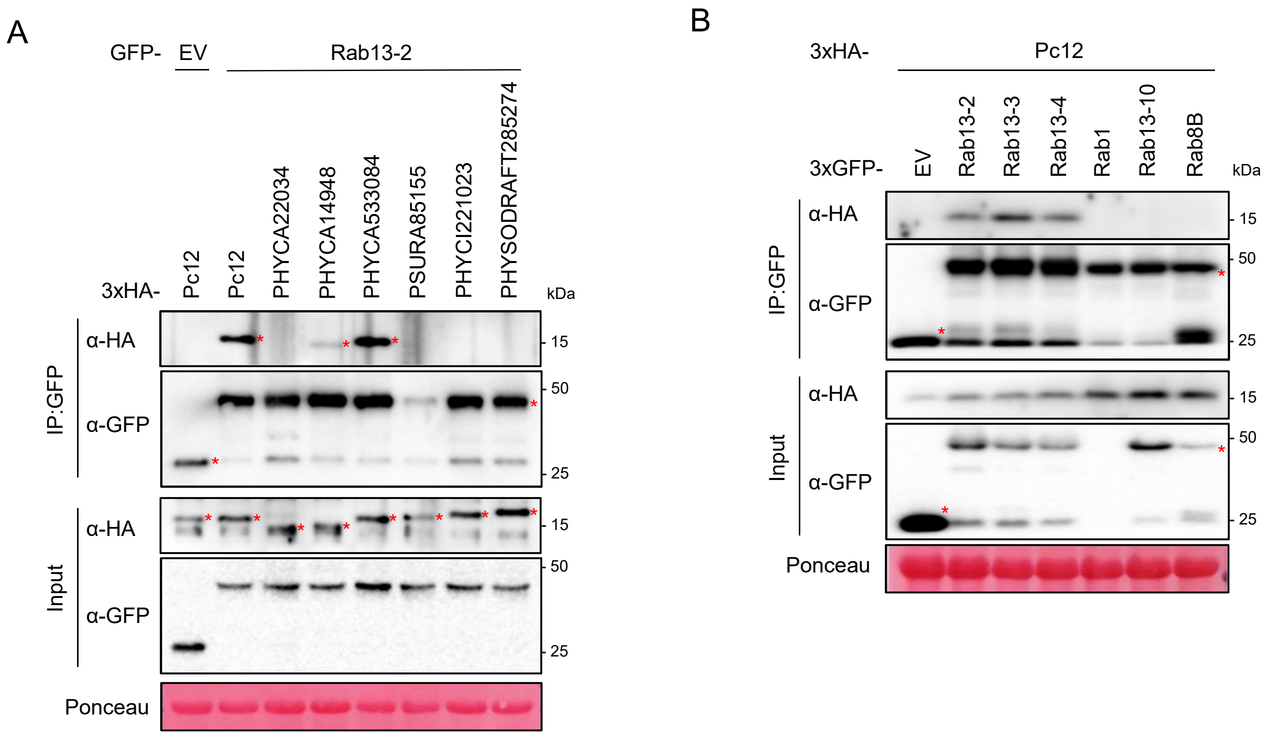

Figure S4. Co-IP with Pc12 homologs and Rab proteins from *N.benthamiana*.
(A) Rab13-2 interacts with Pc12 homologs that induce cell death. GFP- Rab13-2 and 3xHA-Pc12 were transiently expressed in plants. Leaf was sampled at 30 hpi. Total protein extracts were subjected to co-IP using anti-GFP agarose beads (3 experimental replicates).
(B) Pc12 interacts with Rab13-2, Rab13-3, and Rab13-4. Leaf was transiently expressed with 3xHA-Pc12 and GFP-Rab proteins and sampled at 30 hpi (3 experimental replicates).

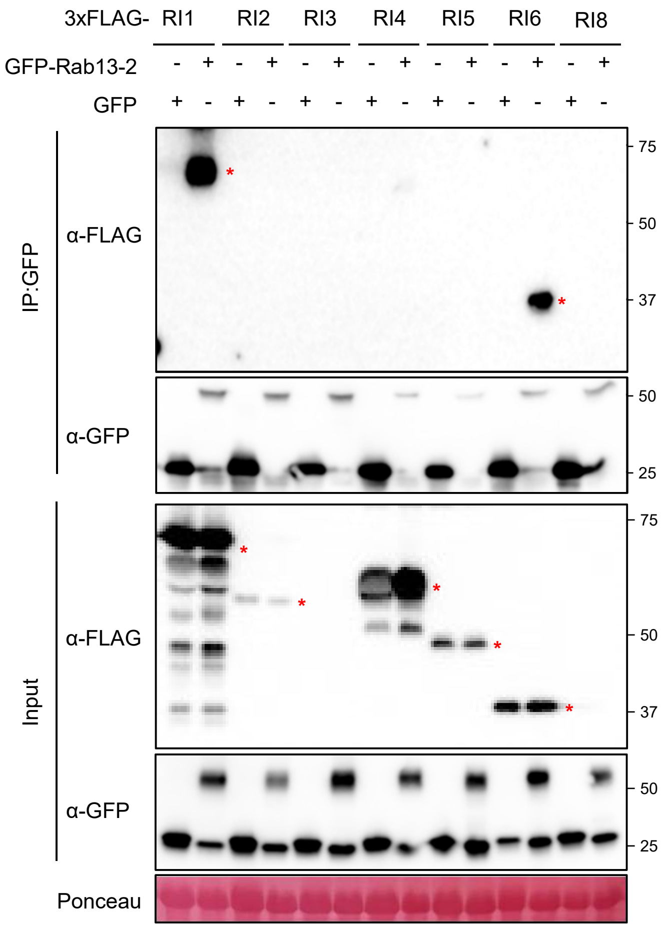

Figure S5. Screening of Rab13-2 interactor candidates from the STRING database
Leaf co-expressing Rab13-2 with interactor candidates in Table S4 was sampled at 48 hpi, and total protein extracts were subjected to co-IP using anti-GFP agarose beads. (3 experimental replicates)

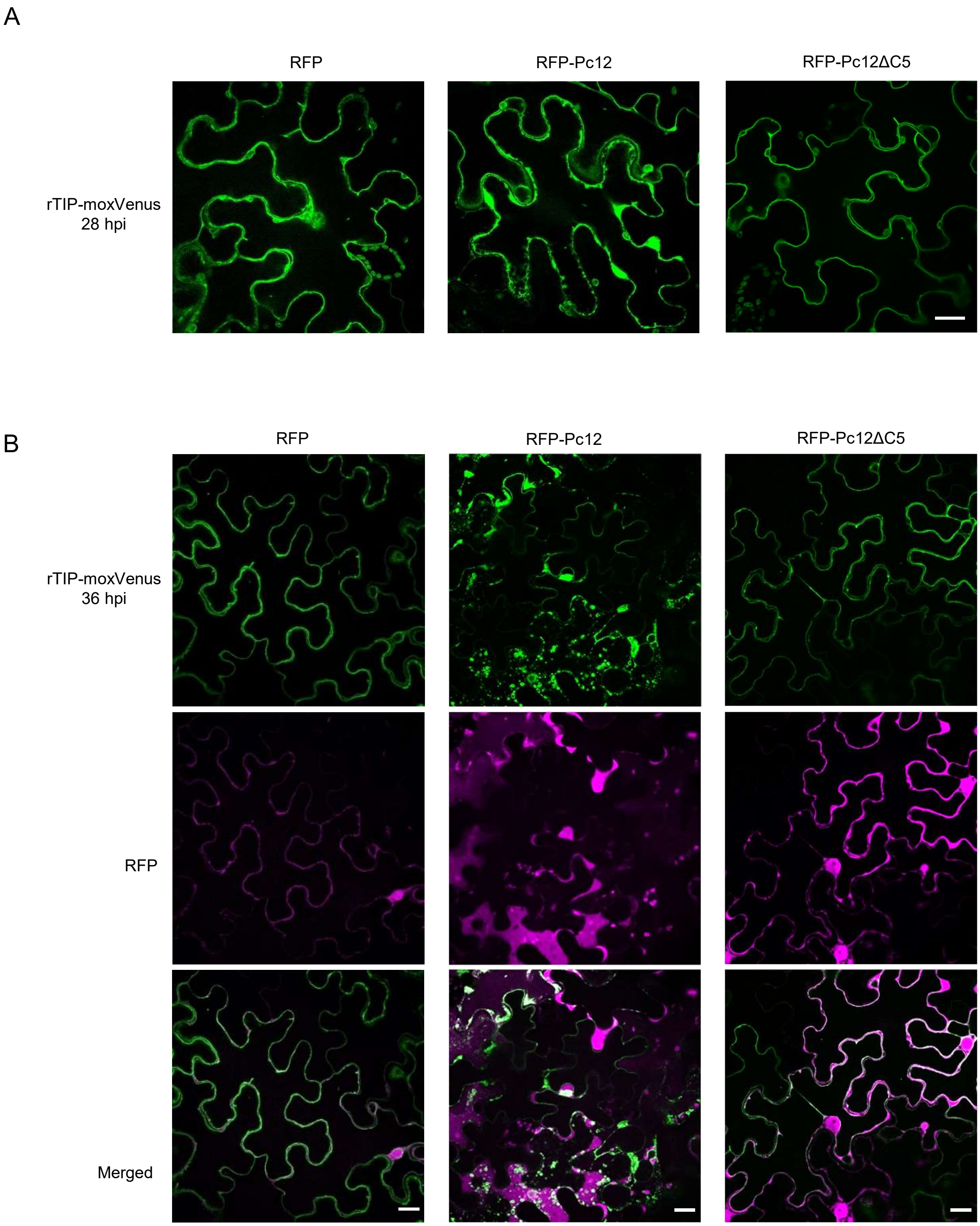
A
RFP
RFP-Pc12
RFP-Pc12ΔC5
rTIP-moxVenus
28 hpi
B
RFP
RFP-Pc12
RFP-Pc12ΔC5
rTIP-moxVenus
36 hpi
RFP
Merged

Figure S6.

Figure S6. Intact vacuolar targeting pathway under Pc12.
(A-B) γ-TIP-moxVenus was transiently expressed in *N. benthamiana*. Since the trafficking of the tonoplast marker takes longer time than that of other proteins, the tonoplast marker was infiltrated 2 days before RFP, RFP-Pc12, or RFP-Pc12ΔC5. Images were taken 28 h, and 36 h after spraying with 1% ethanol. Z-images for sections thicker than 3 μm in 0.75 μm step size were acquired using a laser scanning confocal microscope. Images of the corresponding sections have been processed to improve the brightness for the clarity. This processing does not change the conclusions drawn from the images. moxVenus was pseudo-colored to green. More than 12 images were acquired from 3 independent experiments. Scale bars, 20 μm.

Table S1. Pc12 family in *P. capsici* isolates genome

Oomycetes	Homologs
P. capsici (LT1534)	29
P. capsici (CPV-219)	12
P. capsici (CPV-262)	8
P. capsici (CPV-267)	8
P. capsici (CPV-270)	11
P. capsici (CPV-277)	11
P. capsici (CPV-302)	14
P. capsici (KPC-7)	11
P. capsici (MY-1)	12
P. capsici (JHAI1-7)	12
P. ramorum (Pr102)	2
P. sojae (P6497)	1
P. cinnamomi (CBS 144.22)	1
P. infestans (T30-4)	0

Table S2. Candidates targeted by Pc12 in Mass spectrometry analysis

No.	Accession	# PSMs	Description
1	Niben101Scf07576g00030.1	82	ADP-ribosylation factor-like protein 3
2	Niben101Scf09596g00001.1	63	Ras-related protein Rab-13
3	Niben101Scf01349g03010.1	53	Acetylglutamate kinase
4	Niben101Scf00302g01002.1	37	Fructose-bisphosphate aldolase
5	Niben101Scf01959g04001.1	35	Thioredoxin superfamily protein
6	Niben101Scf03572g04014.1	28	nucleotide binding protein, putative [Ricinus communis] gb |EEF34442.1|
7	Niben101Scf11689g02005.1	27	Aquaporin-like superfamily protein
8	Niben101Scf00635g01010.1	26	BnaA06g33680D [Brassica napus]
9	Niben101Scf00369g08005.1	26	Rubber elongation factor protein
10	Niben101Scf00779g00005.1	19	Uroporphyrinogen decarboxylase
11	Niben101Scf12270g02001.1	18	30S ribosomal protein S11
12	Niben101Scf00288g16005.1	15	40S ribosomal protein S4
13	Niben101Scf01899g07007.1	10	isopropylmalate dehydrogenase 2
14	Niben101Scf08182g01002.1	9	Proteasome activator subunit 4
15	Niben101Scf03138g01010.1	8	Chaperone protein DnaJ
16	Niben101Scf02730g01006.1	7	Peptidyl-prolyl cis-trans isomerase-like 1
17	Niben101Scf16888g00015.1	7	triosephosphate isomerase
18	Niben101Scf01189g04012.1	7	glutamine amidotransferase [Desulfovibrio sp. A2] gb|EGY24267.1| intracellular protease, PfpI family protein [Desulfovibrio sp. A2]
19	Niben101Scf07116g00002.1	6	serine hydroxymethyltransferase 3
20	Niben101Scf09172g01011.1	5	Histidine--tRNA ligase
21	Niben101Scf03114g07016.1	5	septum site-determining protein (MIND)
22	Niben101Scf04417g00001.1	5	Phosphate import ATP-binding protein PstB
23	Niben101Scf09178g01017.1	4	Cysteine desulfurase
24	Niben101Scf20904g00016.1	4	23S rRNA (uracil(1939)-C(5))-methyltransferase RlmD
25	Niben101Scf07767g00008.1	3	Exportin-T
26	Niben101Scf09260g00005.1	3	oxidoreductase, 2OG-Fe(II) oxygenase family protein

Table S3. Sequences of Rab proteins in *N. benthamiana*

Accession number	Sequence
Niben101Scf03781g00001.1_RABB1b	MDSCFLRHPVEQLRMLRKLLLRFFKRSKKVFLMSPTRVNFGSDESGCLDWGVVFDLDKVVLFFSGMNIII
Niben101Scf00726g00012.1_RABA1c	MDYEFHVRILLSAYGRGKVKAKSINHICFTNTEPWLEKLQTRTRPMVEGMLKIGNKIGGKKEVASSLN
Niben101Scf00822g01009.1_Rab-8B	MAVVSPLAKYKLVFLGDQSVGKTSIITRFMYDKFDTTYQATIGIDFLSKTMYLEDRTVRLQLWDTAGQER
Niben101Scf02386g01004.1_Rab-8B	MNPEYDYLFKLLLIGDSGVGKSCLLLRFTDDTYLESYISTIGVDFKIRTVEQDGKTIKLQIWDTAGQERFRTI
Niben101Scf16705g00001.1_Rab-8B	MSNEYDYLFKLLLIGDSSVGKSCLLLRFADDSYVDSYISTIGVDFKIRTVELDGKTIKLQIWDTAGQERFRTI
Niben101Scf00783g05025.1_Rab-8B	MAAVSPLAKYKLVFLGDQSVGKTSIITRFMYDKFDTTYQATIGIDFLSKTMYLEDRTVRLQLWDTAGQER
Niben101Scf02351g02003.1_Rab-8B	HNTFIYYSLPRSPSSLRNSSTMSYAYLFKYIIIGDTGVGKSCLLLQFTDKRFQPVHDLTIGVEFGARMITIDN
Niben101Scf11208g00003.1_Rab-8B	MAYEYLMKFIIVGETGVGKTCIMNQFTEKRFNPVYDVTIGAEYGSKIITVHKKQIKLQIWDTAGQEKFRSIT
Niben101Scf05173g00012.1_Rab-8B	MNPEYDYLFKLLLIGDSGVGKSCLLLRFADDTYQESYISTIGVDFKIRTVEQDGKTIKLQIWDTAGQERFRT
Niben101Scf14778g00018.1_Rab-8B	MSYDYLFKYIIIGDTGVGKSCLLLQFTDKRFQPVHDLTIGVEFGARMVTIDSRPIKLQIWDTAGQESFRSIT
Niben101Scf01625g01009.1_Rab-8B	MSYAYLFKYIIIGDTGVGKSCLLLQFTDKRFQPVHDLTIGVEFGARMIAIDNKPIKLQIWDTAGQESFRSIT
Niben101Scf00046g05014.1_Rab-8B	MSYDYLFKYIIIGDTGVGKSCLLLQFTDKRFQPVHDLTIGVEFGARMESFRSITRSYYSGAAGALLVYDITR
Niben101Scf05032g00004.1_Rab-8B	MIAQGTWAVIPVRCKLYIVFGVEQKSGREHYKLFFVNFASAGFLEALADELGIPFLETSAKDSINVEQAFLT
Niben101Scf01746g03002.1_Rab-8B	MAAVSPLAKYKLVFLGDQSVGKTSIITRFMYDKFDTTYQATIGIDFLSKTMYLEDRTVRLQLWDTAGQER
Niben101Scf01328g01014.1_Rab-8B	MSYAYLFKYIIIGDTGVGKSCLLLQFTDKRFQPVHDLTIGVEFGARMITIDNKPIKLQIWDTAGQESFRSIT
Niben101Scf01517g08034.1_Rab-8B	MSYAYLFKYIIIGDTGVGKSCLLLQFTDKRFQPVHDLTIGVEFGARMITIDNKPIKLQIWDTAGQESFRSIT
Niben101Scf07984g02016.1_Rab-8B	MSYAYLIKYIIIGDTGVGKSCLLLQFTDKRFQPVHDLTIGVEFGARMITIDNKPIKLQIWDTAGQESFRSITR
Niben101Scf01226g01018.1_Rab-8A	MHIFERLLSWLEDILQQRDTTVIVVGNKCDAKEIIAEEGEEFAKSIGYLFIEVSAKITVIVAAVFDKTTERICEK
Niben101Scf09317g00006.1_Rab-8A	MNPEYDYLFKLLLIGDSGVGKSCLLLRFADDSYLESYISTIGVDFKIRTVEQDGKTIKLQIWDTAGQERFRT
Niben101Scf08179g02014.1_Rab-8A	MSHTLSNTLLSVIFRRRSLFPLFPAVRNRNIPVPFALWISQLRKIMNPEYDYLFKLLLIGDSGVGKSCLLLRF
Niben101Scf02197g00001.1_Rab-8A	MAYEYLMKFIIVGETGVGKTCIMNQFTEKRFDPVYDVTIGAGYGSKIITVHTKQIKLQIWDTAGQEKFRSI
Niben101Scf08293g02003.1_Rab-8A	MNPEYDYLFKLLLIGDSGVGKSCLLLRFADDSYLDSYISTIGVDFKIRTVEQDGKTMKLQIWDTAGQERF
Niben101Scf01942g04002.1_Rab-8A	MSMRRRTLLKVIVLGDSGVGKTYVHKKFSQQYKATIGADFVTKELQIDDRLVTLQIWDTAGQERFQSLG
Niben101Scf01993g05016.1_Rab-8A	MSNEYDYLFKLLLIGDSSVGKSCLLLRFADDSYVDSYISTIGVDFKIRTVELDGKTIKLQIWDTAGQERFRTI
Niben101Scf01188g09003.1_Rab-8	MNPEYDYLFKLLLIGDSGVGKSCLLLRFADDTYLESYISTIGVDFKIRTVDQDGKTIKLQIWDTAGQERFRT
Niben101Scf00143g02012.1_Rab-8	MSNEYDYLFKLLLIGDSSVGKSCLLLRFADDSYVESYISTIGVDFKIRTVELDGKTIKLQIWDTAGQERFRTI
Niben101Scf02407g13010.1_Rab-8	MNPEYDYLFKLLLIGDSGVGKSCLLLRFAKIRTVEQDGKTIKLQIWDTAGQERFRTITSSYYRGAHGIIIVYD
Niben101Scf00428g12008.1_Rab-8	MNPEYDYLFKLLLIGDSGVGKSCLLLRFADDSYLESYISTIGVDFKIRTVEQDGKTIKLQIWDTAGQERFRT
Niben101Scf05437g05001.1_Rab-8	MTTAYYRGAMGILLMMYDVTDESSFNNIIRNIEQHPTSKGQALAAEYGIKFFETNWCKDKSECPTCFLLN
Niben101Scf05057g04004.1_Rab-7B	MSMRRRTLLKVIVLGDSGVGKTSLMNQYPSLPHSLIIGGHFYLFIKFTNWQRYVHKKFSQQYKATIGAD
Niben101Scf10036g05001.1_Rab-7a	MAARRRMLLKVIILGDSGYVNRKFSNQYKATIGADVLTKEIQFEDRTRCHFMMLQRASPSDPENFPFVV
Niben101Ctg11584g00002.1_Rab-6C	RTERGSDVIIVLVGNKTDVVDKRQVISFLLRMSSFLFILKLHDPKNMKLNIAASILKDCCCVTWDGGTIIH
Niben101Scf00648g00003.1_Rab-6B	MGCASSVADRNSGRAAGLNPDNGGAVDPKNLKVKLVLLGDSGVGKSCIVLRFVRGQFDPTSKVTVGA
Niben101Scf03215g02001.1_Rab-6A	MVKHLLLCYRRMFKLNKKRKIICKVSILQKYVKSLWNQLVGCCMGKSIKYRKLSRQHSSSTTTTTTAAPP
Niben101Scf29276g00003.1_Rab-6A	MGCASSVADRNSGRAAGLNPDNGGAVDPKNLKVKLVLLGDSGVGKSCIVLRFVRGQFDPTSKVTVGA
Niben101Scf00140g02016.1_Rab-6	MAVVSPLAKYKLVFLRDQSVGKTSIITRFMYDKFDTTYQIAAALPGMEALSSTKGEDMVDVNLKPTTNT
Niben101Scf07070g02007.1_Rab-6	MKSESLHRDLLEITSTMTLVFFRREIFNHLASWLEDVRQHANPNMTIMLVGNKSDPSHRRAVSKEEGEH
Niben101Scf04339g04003.1_Rab-5A	MRHYQLCRKVVQVNTIRWKIVEKVFIFRKFIWERIMVCSTGNSVRYRRLSTKVSEELLTVESSDETLISGYES
Niben101Scf02197g00002.1_Rab-4B	MAYEYRIKFIIVGETGVGKTRLLNQFTEKQFNPVYNVTIGAEYRSKIITIDKKPIKSQIWDTAGQQKFRSITK
Niben101Scf01683g03013.1_Rab-4A	MLQELRYFEIAFLLRPLAMRVYYRDAAGVLLLYGVTKKHSFERLSNWLEDILQQYNTTEGDEFAMSNRYL
Niben101Scf17294g00004.1_Rab-4A	MLTVGGKRLKLTIWDTAGQERFGALTSSYYRGAHGIILVYDVTRRETFTSLSETWARDIKFYSTNPECIKM
Niben101Scf20000g00005.1_Rab-43	MKVVTIIVGNKCNLKDAGEVTTAERNSLAEAQDSSTITVAFQRVVKEIYNFEPEEHSIARTCLMDVAEPTM
Niben101Scf07639g06004.1_Rab-4	MAPVSALAKYKLVFLGDQSVGKTSIITRFMYDKFDNTYQATIGIDFLSKTMYLEDRTVRLQLWDTAGQE
Niben101Scf28369g01004.1_Rab-4	MVVSNCFYYFLLIFISRMIFIRIFLNLVILLLCRRDIFNHLASWLEDGREHGNPNMTIMLVGNKGDLSHRR
Niben101Scf10366g00013.1_Rab-3D	MATESDSSPNDNSQNQAAKDNLNSKPEAAREESPTSVFVNSVPVREDQVQNAIKFLSHPRVRGSPVM
Niben101Scf09456g00016.1_Rab-3B	MIHILKAILAPIGVDFWDTAGQERFRTITSCYYRGAHGIIVVYDVTDQENFNNVRQWLSEIDRYASDNVN
Niben101Scf02914g02029.1_Rab-3A	MTKRHTFERISSWLEDVRQQGDTTVIVMAKKCDAGETIRVVSAEEGEEFIESNGCLFIEAFVKTTVNTATT
Niben101Scf06641g01013.1_Rab-38	MKNFTNLCFINCKYRYVFKKFRQQYKATIGADFATKQVEIDERVATLQIWDTAGQERFHSLGVAFYRGA
Niben101Scf16332g01002.1_Rab-33	MMFWRERERETKEQNGGPPCGQVRVLVVGDSGVGKTSLVHLILNGSSIARPPQTIGCTVGVKHTTGGN
Niben101Scf07499g00003.1_Rab-31	MAPVSALAKYKLVFLGDQSVGKTSIITRFMYDKFDNTYQATIGIDFLSKTMYLEDRTVRLQLWDTAGQE
Niben101Scf10476g02018.1_Rab-31	MAPVSALAKYKLVFLGDQSVGKTSIITRFMYDKFDNTYQATIGIDFLSKTMYLEDRTVRLQLWDTAGQE
Niben101Scf00858g04011.1_Rab-2B	MDAGTRHTFERLSSWLEDIRQQGDTTVIVVGNKCDDGGNIRAINAEECEEFAKSNGCLVIEAFAIEEMDE
Niben101Scf01241g02013.1_Rab-2B	MTIMLVGNKSDLSHSRAVSKEEGEQFAKENGLLFLEASARTAQNVEETAARILQKIQEGVFDVSNESSGI
Niben101Scf01253g01004.1_Rab-2A	MLVYDLTKRQSFDHMARWLEELRGHADKNIVIMIIEIYRIISKNTLTANPGADYGKYSKSLKGTRIIVPGQ
Niben101Scf14669g00004.1_Rab-2A	MVDNEEGDQYAKENGLLFFETSAKTAQNINELFHVIAKRLASAAPAHLGGMKLGNRTEKGVRRLFCCS
Niben101Scf17221g00006.1_Rab-2A	WWEIRECDAGKKNRTVNAEEGKEFAKSNRCLFIEASAKTTMNVAAAFEKTAVRKCEKMGYRDVEEMNE
Niben101Scf05118g08005.1_Rab-2A	MTVSAEEGEEFAKSNGYLFVEASAKTAMNVAVAFDKTTERICEKMGYGDVEEVDEALGINKWNWKRKV
Niben101Scf03775g00001.1_Rab-2A	MASTGHNNLNAKLVLLGDMGAGKSSLVIRFVKGQFLEFQESTIGAAFFSSTVSVNNATVKFEIWDTAG
Niben101Scf02543g04012.1_Rab-26	MTKRKSINYRQLSHNQSFPTTTTTTRNMASPPSSAVASSESGFSGSLASTCCCDHSKDSSEDVVALKISLL
Niben101Scf02892g01013.1_Rab-26	MESFNNVKQWLNEIDRYANESVCKLLVGNKCDLVENKVVDTQTGKASANELGIPFLEKSAKDPINVDRT
Niben101Scf11823g00020.1_Rab-26	MATTGQSNSSYDLSFKILLIGDSGVGKSSLLVSFISNVVDDLAPTIGVDFKIKTLTVGGKRLKLTIWDTVYD
Niben101Scf03878g00004.1_Rab-26	MASPLSSAVASSESGFSGSLASTCCCDHSKDSSDDTKYVGKEKAADEDFSTKGLNQMDKTLCVKGTRIS
Niben101Scf00679g08006.1_Rab-26	MTKRLLLCYRRMLHLNMKRKIICKFTILHKYFSSFWNKILACWIGKSINYRQLSHNQSFPTTTTTTRNMTS
Niben101Scf00457g05011.1_Rab-26	MATTGQSNSSYDLSFKILLIGDSGVGKSSLLVSFISNAVDDLAPTIGVDFKIKTLTVGRKRLKLTIWDTVYD
Niben101Scf02568g02007.1_Rab-26	MTIMLIGNKCDLAHRRAVSTEEQFAKENGLIFMEASAKTAQNVEEAFIRTASTIYIRKYRMECLMYQVRV
Niben101Scf06085g00009.1_Rab-25	MAFRSEEAQTEDYLFKIVLIGDSAVGKSNLLARFARDEFYPNSKSTIGVEFQTQKLDINGKEVKAQIWDTA
Niben101Scf05032g01011.1_Rab-25	MARRAEEEYDYLFKIVLIGDSGVGKSNLLSRFTRNEFLLESKSTIGVEFATRTLQVEGRTVKAQIWDTAGQ
Niben101Scf02892g01023.1_Rab-25	MARRAEEEYDYLFKIVLIGDSGVGKSNLLSRFTRNEFLLESKSTIGVEFATRTLQVEGRTVKAQIWDTAGQ
Niben101Scf01234g00006.1_Rab-25	MAFRSEEAQTEDYLFKIVLIGDSAVGKSNLLARFARDEFYPNSKSTIGVEFQTQKLDINGKEVKAQIWDTA
Niben101Scf02911g06015.1_Rab-25	MELHSEEEKKEDYLFKIVLIGDSAVGKSNLLARFARDEFYPNSKSTIGVEFQTQKMDINGKEVKAQIWDTA
Niben101Scf07314g03002.1_Rab-25	MGAYRTDEDYDYLFKVVLIGDSGVGKSNLLSRFTKNEFSQESKSTIGVEFATRTIHVDDKIVKAQIWDTA
Niben101Scf18551g00010.1_Rab-25	MASGYGDASQKIDYVFKVVLIGDSAVGKSQILARFARNEFSLDSKATIGVEFQTRTLVIQHKSVKAQIWD
Niben101Scf01763g02015.1_Rab-25	MAEEEVGEEYLFKIVVIGDSAVAKSNLLSRFARDEFDHNSKATIGVEFQTQVVEIDGKEIKAQVWDTAGQ
Niben101Scf02414g02002.1_Rab-25	MSNLYGDYNQKIDYVFKIVLIGDSAVGKSQLLARFARNEFSLDSKATIGVEFQTKTLIIDHKTVKAQIWDT
Niben101Scf06023g03018.1_Rab-25	MSNLYGDYNQKIDYVFKIVLIGDSAVGKSQLLARFARNEFSLDSKATIGVEFQTKTLIIDHKTVKAQIWDT
Niben101Scf00270g12012.1_Rab-25	MDSSDNESGEEYLFKIVIIGDSAVGKSNLLTRYARNEFNLHSKATIGVEFQTQTLEIDGKEVKAQIWDTAG
Niben101Scf10268g00002.1_Rab-25	MGGYRTEDDYDYLFKVVLIGDSGVGKSNLLSRFTRNEFSLESKSTIGVEFATKSLTVDTKVIKAQIWDTAG
Niben101Scf38085g00003.1_Rab-25	MGNRVDHEYDYLFKIVLIGDSGVGKSNILSRFTRNEFCLESKSTIGVEFATRTLQVEGKTVKAQIWDTAG
Niben101Scf11356g00014.1_Rab-25	MAEEEVGEEYLFKIVVIGDSAVGKSNLLSRFARDEFDHNSKATIGVEFQTQVVEIDGKEIKAQVWDTAGQ
Niben101Scf00985g06001.1_Rab-25	MSKNSYGDYNQKIDYVFKIVLIGDSAVGKSQLLARFARNEFSLNSKATIGVEFQTKTLLVDNRTVKAQIW
Niben101Scf32882g00003.1_Rab-25	MAYKVDHEYDYLFKIVLIGDSGVGKSNILSRFTRNEFCLESKSTIGVEFATRTLQAQIWDTAGQERYRAITS
Niben101Scf05617g01020.1_Rab-25	MNQEMSGVIVADQKQQQEMKGVNGGVHEDIKIDYVFKVVVIGDSAVGKTQVLSRFAKNEFCFDSKST
Niben101Scf00143g02013.1_Rab-25	MARRPDEEYDYLFKIVLIGDSGVGKSNLLSRFTRNEFCLESKSTIGVEFATRTLQVEGRIIKSQIWDTAGQE
Niben101Scf11362g01012.1_Rab-25	MGAYRADDDYDYLFKVVLIGDSGVGKSNLLSRFSRNEFSLESKSTIGVEFATRSIRVEDKVVKAQIWDTA
Niben101Scf05372g00006.1_Rab-25	MANRVDHEYDYLFKIVLIGDSGVGKSNILSRFTRYEFCLESKSTIGVEFATRTLQVEGKTVKAQIWDTAGQ
Niben101Scf03779g08009.1_Rab-25	REVEMAYKVDHEYDYLFKIVLIGDSGVGKSSILSRFTRNEFCLESKSTIGVEFATRTLQVEGKTVKAQIWDT
Niben101Scf03023g02007.1_Rab-25	MGAYRADEDYDYLFKVVLIGDSGVGKSNLLSRFTKNEFSQESKSTIGVEFATRTIHVDDKIIKAQIWDTAG
Niben101Scf03577g00001.1_Rab-25	MDSSDDDIEEYLFKIVIIGDSAVGKSNLLSRYARDEFNLHSKATIGVEFQTQVLEINGKEVKAQIWDTAGQ
Niben101Scf01119g01007.1_Rab-25	MAGGYRAEDDYDYLFKLVLIGDSGVGKSNLLSRFSRNEFNLESKSTIGVEFATRSIRVDDKIVKAQIWDTA
Niben101Scf05811g03003.1_Rab-25	MGNRVDHEYDYLFKIVLIGDSGVGKSNILSRFTRNEFCLESKSTIGVEFATRTLQVEGKTVKAQIWDTAG
Niben101Scf00644g00011.1_Rab-25	MSIMGDSFDEECDYLFKAVLIGDSAVGKSNLLSRFAKDQFHLDSKPTIGVEFAYRNIRVGDKLIKAQIWD
Niben101Scf00192g01023.1_Rab-25	MGAYRADDDYDYLFKVVLIGDSGVGKSNLLSRFSRNEFSLESKSTIGVEFATRSIRVEDKVVKAQIWDTA
Niben101Scf00463g00018.1_Rab-25	MAGYRADDEYDYLFKLVLIGDSGVGKSNLLSRFTKNEFNLESKSTIGVEFATKSLNIDNKVIKAQIWDTA
Niben101Scf05391g01016.1_Rab-25	MGAYRSEEDYDYLFKVVLIGDSGVGKSNLLSRFTKNEFSQQSKSTIGVEFATRTIHVDDKIIKAQIWDTAG
Niben101Scf06525g01002.1_Rab-25	MDSSDDDIEEYLFKIVIIGDSAVGKSNLLSRYARDEFNLHSKATIGVEFQTQVLEINGKEVKAQIWDTAGQ
Niben101Scf00528g01022.1_Rab-25	MLVYDITKLQSFDHVARWLEELRGHADKNIVIMLVGNKTDLGSLRAVPTEDAKECAEKENLFFIETSALEA
Niben101Scf10438g02002.1_Rab-25	MASGYGDPSQKIDYVFKVVLIGDSAVGKSQILARFARNEFSLDSKATIGVEFQTRTLVIQHKSVKAQIWD
Niben101Scf09191g02003.1_Rab-25	MGAYRADDDYDYLFKVVLIGDSGVGKSNLLSRFTRNEFSLESKSTIGVEFATRSIRVDDKVVKAQIWDTA
Niben101Scf05351g00010.1_Rab-25	MANRVDHEYDYLFKIVLIGDSGVGKSNILSRFTRNEFCLESKSTIGVEFATRTLQVEGKTVKAQIWDTAG
Niben101Scf01607g01010.1_Rab-25	MGAYRADDDYDYLFKVVLIGDSGVGKSNLLSRFTRNEFSLESKSTIGVEFATRSIRVDDKVVKAQIWDTA
Niben101Scf12789g00027.1_Rab-25	MAEEDVGEEYLFKIVVIGDSAVGKSNLLSRFARDEFDHNSKATIGVEFQTQVVEIDGKEIKAQVWDTAGQ
Niben101Scf02653g04021.1_Rab-25	MAYKVDHEYDYLFKIVLIGDSGVGKSNILSRFTRNEFCLESKSTIGVEFATRTLQVEGKTVKAQIWDTAGQ
Niben101Scf02216g02002.1_Rab-25	MSKLYGGVFNQKIDYVFKVVLIGDSAVGKSQLLARFSRNEFSLDSKATIGVEFQTRTLDIDHKTVKAQIW
Niben101Scf00581g01016.1_Rab-25	MGGYRTEDDYDYLFKVVLIGDSGVGKSNLLSRFTRNEFSLESKSTIGVEFATKSLTVDTKVIKAQIWDTAG
Niben101Scf09987g02004.1_Rab-25	MAYKVDHEYDYLFKIVLIGDSGVDLPEMNFVWNLSLPLASNLQQGPFRYRAITSAYYRGAVGALLVYDIT
Niben101Scf20512g01001.1_Rab-25	MAGGYRAEDDYDYLFKLVLIGDSGVGKSNLLSRFSRNEFNLESKSTIGVEFATRSIRVDDKIVKAQIWDTA
Niben101Scf09620g00003.1_Rab-25	MNQEMSGVIVEDQKQKQEMKGVNEGVHEDIKIDYVFKVVLIGDSAVGKTQVLSRFAKNEFCFDSKSTI
Niben101Scf10043g00010.1_Rab-25	MLIGNKSDLRHLVAVSTDEAKGLAEREALYFMETSALEATNVENAFTEVLTQIYRIVSKKAVEAGDEGATS
Niben101Scf05566g06004.1_Rab-25	MASGGGYGDASQKIDYVFKVVLIGDSAVGKSQILARFARNEFSLDSKPTIGVEFQTRTLVIQHKSVKAQI
Niben101Scf01376g04023.1_Rab-25	MDSSDDESGEEYLFKIVIIGDSAVGKSNLLTRYARNEFNLHSKATIGVEFQTQTLEIDGKEVKAQIWDTAG
Niben101Scf01993g05015.1_Rab-25	MARRPDEEYDYLFKIVLIGDSGVGKSNLLSRFTRNEFCLESKSTIGVEFATRTLQVEGRIIKSQIWDTAGQE
Niben101Scf01466g00001.1_Rab-25	MANGGGYGDASQKIDCVFKVVLIGDSAVGKSQILARFARNEFSLDSKATIGVEFQTRTLVIQHKSVKAQI
Niben101Scf01784g00010.1_Rab-24	MALTKGRSTTYGQYSQSYIVLNLTFHDENTNLSSAGLSLSFLLVHTLFFYFLGFYWMATRNKNIQAKLVL
Niben101Scf07253g02019.1_Rab-21	MASSGHNNLNAKLVLLGDMGAGKSSLVIRFVKGQFLEFQESTIGAAFFSSTLAVNNATVKFEIWDTAG
Niben101Scf17301g00001.1_Rab-21	MLMFLFGWPIFMRTSPLELCQHLTTLLHWCWSADVSIWMAHLHEDFSIGALPAFDHLIALVLVFDISDLS
Niben101Scf07008g01002.1_Rab-1D	MASRRRMLLKVIILGDSGYVNRKFSNQYKATIGADFLTKEVQFEDRLYTLQIWDTAGQERFQSLGVAFYF
Niben101Scf06726g00033.1_Rab-1D	MPSRRRTLLKVIILGDSGYVNKKFSNQYKATIGADFLTKEVQFEDRLFTLQIWDTAGQERFQSLGVAFYR
Niben101Scf01374g03034.1_Rab-1C	MASRRRMLLKVIILGDSGYVNRKFSNQYKATIGADFLTKEVQFEDRLYTLQIWDTAGQERFQSLGVAFYF
Niben101Scf04778g00002.1_Rab-1C	MRRKDRSPPKMPSRRRTLLKVIILGDSGVGKTSLMNQYVNKKFSNQYKATIGADFLTKEVQFEDRLFTL
Niben101Scf00449g05005.1_Rab-1B	MLDELSSLGIIIMEKSNKLYTLCTIYCFLSIFSRPCIFLFSSKFTGFLCFYRIDFKIRTIELDGKRIKLQIWDTAG
Niben101Scf07650g01021.1_Rab-1A	MAAPPARARADYDYLIKLLLIGDSGVGKSCLLLRFSDGSFTTSFITTIGIDFKIRTIELDGKRIKLQIWDTAG
Niben101Scf14010g01019.1_Rab-1A	MTPLTRIINIFEFSGVGKSCLLLRFSDGSFTTSFIITIGIDFKIRTIELDGKRIKLQIWDTAGQERFRTITTGIHLD
Niben101Scf35013g00007.1_Rab-19	MDINGKEVKAQIWDTAGQERFRAVTSAYYRSAVGALLVYDNSRRLTFDSIGRWLNELQTLDSSNVASA
Niben101Ctg15946g00001.1_Rab-17	MRAYYRDVAGALLVYNVIKRYTFERLPSWLEDILPQYDTTWWGIRKCDAGENFRTISANERGEFAKSNG
Niben101Scf28100g00001.1_Rab-17	MATRRNKNIQAKLVLLGDMGTGKTSLVLRFVKGQFFDFQEPTIGAAFFTQLLSLNEATIKFDIWDTAGQ
Niben101Scf02976g01016.1_Rab-14	MVMALAGNKADLLDARKVAAEEAQTYAQENGLFFMETSAKTASNVNDIFYEIAKRLPRLQPAQNPSGN
Niben101Scf14979g00002.1_Rab-14	AVEALLIYDVTSRHTFEWLSSWLEDILQQHDTTWWGITECDAGENIRTVTAEESEEFTKSNGYLFIEASAET
Niben101Scf01992g00005.1_Rab-14	AYYKDDVRAFLVYNVTKRHTFERLSSWLEDILQQNDRTWWGINEGDAIKYIWTVSAEEGEKFAKSSGCL
Niben101Scf03131g00002.1_Rab-14	MASTGHNNLNAKLVLLGDMGAGKSSLVIRFVKGQFLEFQESTIGAAFFSSTVSVNNATVKFEIWDTAG
Niben101Scf00684g00002.1_Rab-13	MAAPPARARADYDYLIKLLLIGDSGVGKSCLLLRFSDGSFTTSFITTIGIDFKIRTIELDSKRIKLQIWDTAG
Niben101Scf06078g02029.1_Rab-13	MNPEYDYLFKLLLIGDSGVGKSCLLLRFADDSYLESYISTIGVDFKIRTVEQDGKTIKLQIWDTAGQERFRT
Niben101Scf01006g07002.1_Rab-13	MSKPLRSLIKVIVLGDCGQQQYKATIGADFATKELEIDQRVATLQIWDTAGQERFHSLGVAFYRGADCCILV
Niben101Scf10466g00002.1_Rab-13	MIMDMRKNQNGGNGCYDYSFKILLVGDSGVGKSSLLLSFISQHPPQDLSPTIGVDFKIRMLTVGGKRLK
Niben101Scf00792g01002.1_Rab-13	MIMDMRKNQNGGNGCYDYSFKILLVGDSGVGKSSLLLSFISQHPPQDLSPTIGVDFKIRMLTVGGKRLK
Niben101Scf19059g00005.1_Rab-13	MAAPPARARADYDYLIKLLLIGDSGVGKSCLLLRFSDGSFTTSFITTIGIDFKIRTIELDGKRIKLQIWDTAG
Niben101Scf05709g00001.1_Rab-13	MAARRRMLLKVIILGDSGVGKTSLMNQYVNRKFSNQYKATIGADFLTKEIQFEDRLYTLQIWDTAGQER
Niben101Scf02606g00015.1_Rab-13	MAAPPARARADYDYLIKLLLIGDSGVGKSCLLLRFSDGSFTTSFITTIGIDFKIRTIELDGKRIKLQIWDTAG
Niben101Scf03277g02014.1_Rab-13	MAAPPARARADYDYLIKLLLIGDSGVGKSCLLLRFSDGSFTTSFITTIGIDFKIRTIEFDSKRIKLQIWDTAG
Niben101Scf09596g00001.1_Rab-13	MEPGKSKEGNGKRDEGDHHEDNKKSIAEPPHLLDLKHIFISLFFFPFTLLRSRWRYSPSSFFPEKIQMAVP
Niben101Scf07194g00014.1_Rab-12	MTNLCRKIVHVNIRWKIFDKVSIFREFFRFIWERILICSTGREPVRKYRRLSRRAASPSMEEREEYCGENDPL
Niben101Scf22427g00002.1_Rab-12	MEQQQQEQQQNPPIVDDNSLENKPGIIILGSPNVGKRTLLSRLLSIDSEDAFDSSSNILAYPWTINTKYYK
Niben101Scf12084g01001.1_Rab-12	MMFWRERERETKEQNGGPPCGQVRVLVVGDSGVGKTSLVHLILNGSSVARPPQTIGCTVGVKHTTYGN
Niben101Scf05057g04003.1_Rab-10	MQANPPDPKTFPFILLGNKIDIDGGNSRVVSEKKAKEWCASKGIPYFETSAKEDINVDAAFLSIAKTALAN
Niben101Scf02976g01015.1_Rab-1	MATSGNKNMNAKLVLLGDVGAGKSSLVLRFVKGQFIEFQESTIGAAFFSQTVAVNDATVKFEIWDTAG
Niben101Scf00271g01020.1_Rab-1	MATGGNKNMNAKLVLLGDVGAGKSSLVLRFVKGQFIEFQESTIGAAFFSQTVAVNDATVKFEIWDTAG
Niben101Scf08526g01012.1_Rab-1	MSMRRRTLLKVIVLGDSGVGKTSLMNRYVHKKFSQQYKATIGADFVTKELQIDDRLVTLQIWDTAGQE
Niben101Scf05032g00003.1_Rab-1	MLSSIVYDVTEIESFNNVKQWLNEIDRYANESVCKLLVGNKCDLVENKVVDTQTEKWKIEDEHTLDPTVG
Niben101Scf05032g00006.1_Rab-1	MSNEYDYLFKLLLIGDSSVGKSCLLLRFADDSYVDSYISTIGVDFKIRTAELDGKTIKLQIWDTAGQERFRTI
Niben101Scf02791g01001.1_Rab-1	MEASSSSPEFDYLFKLLLIGDSGVGKSSLLLSFTSDAFEDLSPTIGVDFKVKHVTLGGKKLKLAIWDTAGQ

Table S4. Rab13-2 interactors from STRING

No.	Gene	Description
RI1	REP1	rab escort protein 1 [Nicotiana attenuata]
RI2	PEX7-3	peroxisome biogenesis protein 7 [Nicotiana attenuata]
RI3	RAP2-3	ethylene-responsive transcription factor RAP2-3 [Nicotiana attenuata]
RI4	A0A1J6IQ56	uncharacterized protein LOC109234543 [Nicotiana attenuata]
RI5	A0A314KHE5	deSI-like protein At4g17486 [Nicotiana attenuata]
RI6	PRA1F2	PRA1 family protein F3-like [Nicotiana attenuata]
RI7	SEC6	exocyst complex component sec6 [Nicotiana attenuata]
RI8	PIP5K2	phosphatidylinositol 4-phosphate 5-kinase 2-like [Nicotiana attenuata]

Table S5. Primer sequences used in this study

Primer name	Sequence (5'-3')	Description	Reference
**Cloning**			
Pc12_linker	GgtGgcTcgGgcGgaTCTGgaGgtTcaGgtGgcTcg ATGTTCTCTATC TTCGGAGAGAAGGC	Overexpression with N-terminal epitope tag	
Pc12_LIC_R	GAGGAGAAGAGCCCT TTAGCCCATTTGATGAGCGAGATAC	Overexpression with N-terminal epitope tag	
Pc12dC5_LIC_R	GAGGAGAAGAGCCCTTTAGAGATACTTTCCGTAGCCCAC	Overexpression with N-terminal epitope tag	
Rab13-2	GgaTCTGGAGgcAGC ATGGCCGTTCCACCCG	Overexpression with N-terminal epitope tag	
Rab13-2_LIC_R	GAGGAGAAGAGCCCT TCAAGAGCCACAGCAAGCTG	Overexpression with N-terminal epitope tag	
REP_LIC	CGACGACAAGACCCTATGGATGAAACTTTGTCGTA	Overexpression with C-terminal epitope tag	
REP_LIC_R	GAGGAGAAGAGCCCT GTCCTCGAGCTCTTCGAC	Overexpression with C-terminal epitope tag	
PRA1F2_LIC	CGACGACAAGACCCT ATGACGAATTACGGCACAAT	Overexpression with C-terminal epitope tag	
PRA1F2_LIC_R	GAGGAGAAGAGCCCT AGACGACGGAGGAGCT	Overexpression with C-terminal epitope tag	
Pc12_EcoRI	atgtgtccaaaGAATTCATGTTCTCTATCTTCGGAGAGAAG	pGBKT7 (yeast bait vector)	
Pc12_PstI_R	tttttgcacgcatCTGCAGTTAGCCCATTTGATGAGCGA	pGBKT7 (yeast bait vector)	
Pc12dC5_PstI_R	ATGCGGCCGCTGCAGTTAGAGATACTTTCCGTAGCCCACC	pGBKT7 (yeast bait vector)	
Rab13-2_EcoRI	atgtgtccaaaGAATTC ATGGCCGTTCCACCCG	pGADT7 (yeast prey vector)	
Rab13-2_BamHi_R	GAGCTCGATGGATCC TCAAGAGCCACAGCAAGCTG	pGADT7 (yeast prey vector)	
**qRT-PCR**			
EF1a_qRT	GTATGCCTGGGTGCTTGAC	Nomalization	Heese et al., 2007
EF1a_qRT_R	ACAGGGACAGTTCCAATACCA	Nomalization	Heese et al., 2007
NbSGT1_qF	CCTGCAAAATGCAGATATGAAGTG	Gene silencing	
NbSGT1_qR	TCTGCACTACAGCAGACGC	Gene silencing	
NbNRG1_qF	ACTTCAACCACTTCAGGCGG	Gene silencing	
NbNRG1_qR	GAGATGCGTTCCAGGCTGAT	Gene silencing	
NbADR1_qF	TTTCACAGACACGTCAGCGTC	Gene silencing	
NbADR1_qR	CCGTCGTCAACTCCAATCTTTATAGC	Gene silencing	
NbNRC2_qF	GCTAAGCTTCACAAGGACAAAG	Gene silencing	Oh et al., 2023
NbNRC2_qR	CAGATTGATCTTCATCTTGAAGG	Gene silencing	Oh et al., 2023
NbNRC3_qF	GACGATAAAAATAAATTTGCTCAGTGG	Gene silencing	Oh et al., 2023
NbNRC3_qR	CTGAGCACCTTGGTTGAAATTATC	Gene silencing	Oh et al., 2023
NbNRC4_qF	GAACAGTGGAAAGTGTTGGTGG	Gene silencing	Oh et al., 2023
NbNRC4_qR	GGTCATGAATTCCTTTGACCTC	Gene silencing	Oh et al., 2023
NbEDS1_qF	CAAAGGTGTTCGTTACGATG	Gene silencing	
NbEDS1_qR	CCTGAACCTGCAATTGTAAAAC	Gene silencing	
NbPR1_qF	AATAGGGTAGCGGCCTTTGC	Defense gene	Roshan et al., 2018
NbPR1_qR	CGGCGGCTAGGTTTTCG	Defense gene	Roshan et al., 2018
NbRbohB_qF	CAGTCTCTTCACCATGCCAAAAA	Defense gene	Li et al., 2015
NbRbohB_qR	CCCACAATAGAAGACCCCAACT	Defense gene	Li et al., 2015
NbWRKY8_qF	AACAATGGTGCCAATAATGC	Defense gene	Moon et al., 2016
NbWRKY8_qR	TGCATATCCTGAGAAACCATT	Defense gene	Moon et al., 2016
PcTubulin	CAACAACAGTTCGATGCTAAGAACA	Nomalization	
PcTubulin_R	CCATCTCATCCATACCCTCGCCAG	Nomalization	
PcHmp1_qF	CATGATGGCAGTCATGGTCGGTGAAG	P. capsici gene	Jupe et al., 2013
PcHmp1_qR	TTAGCTAACATTGAGGCGGGCATGCAG	P. capsici gene	Jupe et al., 2013
PcNPP1_qF	CAGCTCCACATCACCAACGGct	P. capsici gene	Jupe et al., 2013
PcNPP1_qR	CTCTTCCCGTTCAAATAGTTC	P. capsici gene	Jupe et al., 2013
Pc12_qF	CGACCGCGGTGAGCA	P. capsici gene	
Pc12_qR	TTAGCCCATTTGATGAGCGA	P. capsici gene	
NtBLP4_qF	AGCTTTGAGCAGTCAACACCAAGT	ER stress gene	
NtBLP4_qR	AAAACGTGCCCGAGTAAGTGGTTC	ER stress gene	
NbCRT_qF	CGAAGAAGAAAAGAAGAGAGAGGAG	ER stress gene	
NbCRT_qR	CATCATCCTTGGACTCCGAG	ER stress gene	
NbbZIP28_qF	TTCCTAGCCGATTGTCTCGGG	ER stress gene	
NbbZIP28_qR	GCTGCTTGATTTCCGGAAAAATTACc	ER stress gene	
NtbZIP60_qF	CCTGCTTTGGTTCCTGGGCATCAT	ER stress gene	
NtbZIP60_qR	AGAAGACCGTGGTTTCTGCTTCGT	ER stress gene	
